# CRISPR/Cas9 RNP-assisted validation of palmarumycin biosynthetic gene cluster in *Lophiotrema* sp. F6932

**DOI:** 10.3389/fmicb.2022.1012115

**Published:** 2022-09-29

**Authors:** Martin Muthee Gakuubi, Kuan Chieh Ching, Madhaiyan Munusamy, Mario Wibowo, Chun Teck Lim, Guang-Lei Ma, Zhao-Xun Liang, Yoganathan Kanagasundaram, Siew Bee Ng

**Affiliations:** ^1^Singapore Institute of Food and Biotechnology Innovation (SIFBI), Agency for Science, Technology and Research (A*STAR), Singapore, Singapore; ^2^School of Biological Sciences, Nanyang Technological University, Singapore, Singapore

**Keywords:** CRISPR-Cas9, ketosynthase domain, *Lophiotrema* sp., palmarumycin, polyketide synthases

## Abstract

*Lophiotrema* is a genus of ascomycetous fungi within the family *Lophiotremataceae*. Members of this genus have been isolated as endophytes from a wide range of host plants and also from plant debris within terrestrial and marine habitats, where they are thought to function as saprobes. *Lophiotrema* sp. F6932 was isolated from white mangrove (*Avicennia officinalis*) in Pulau Ubin Island, Singapore. Crude extracts from the fungus exhibited strong antibacterial activity, and bioassay-guided isolation and structure elucidation of bioactive constituents led to the isolation of palmarumycin C_8_ and a new analog palmarumycin CP_30_. Whole-genome sequencing analysis resulted in the identification of a putative type 1 iterative PKS (iPKS) predicated to be involved in the biosynthesis of palmarumycins. To verify the involvement of palmarumycin (PAL) gene cluster in the biosynthesis of these compounds, we employed ribonucleoprotein (RNP)*-*mediated CRISPR-Cas9 to induce targeted deletion of the ketosynthase (KS) domain in PAL. Double-strand breaks (DSBs) upstream and downstream of the KS domain was followed by homology-directed repair (HDR) with a hygromycin resistance cassette flanked by a 50 bp of homology on both sides of the DSBs. The resultant deletion mutants displayed completely different phenotypes compared to the wild-type strain, as they had different colony morphology and were no longer able to produce palmarumycins or melanin. This study, therefore, confirms the involvement of PAL in the biosynthesis of palmarumycins, and paves the way for implementing a similar approach in the characterization of other gene clusters of interest in this largely understudied fungal strain.

## Introduction

Lack of a versatile genetic engineering system that can be employed across the vast majority of non-model filamentous fungi has been a long-standing challenge toward a broader exploitation of fungal secondary metabolite biosynthetic capabilities (Nødvig et al., 2015; Schuster and Kahmann, 2019). Many of the early genetic engineering systems in filamentous fungi were developed for the model strains such as *Aspergillus oryzae, Aspergillus nidulans, Trichoderma reesei,* and *Neurospora crassa* (Wang et al., 2020; Jiang et al., 2021; Jin et al., 2021). The emergence of clustered regularly interspaced short palindromic repeats (CRISPR)-Cas9 system, a programmable gene editing technology that is convertible with all cell types, has greatly expanded the avenue for genetic manipulation of living organisms including fungi. Up till now, various forms of CRISPR-Cas9 genetic engineering protocols have been established in more than 40 species of filamentous fungi and Oomycetes (Schuster and Kahmann, 2019). Derived from the bacterial adaptive immune system, CRISPR-Cas9 technology is a powerful molecular tool that allows for precise DNA editing and has accelerated the pace of research in filamentous fungi (Deng et al., 2017; Krappmann, 2017; Schuster and Kahmann, 2019). Type II CRISPR-Cas9, the most common of the CRISPR systems consists of two main components: (a) a CRISPR-associated Cas9 endonuclease derived from *Streptococcus pyogenes* and (b) a single-guide RNA (sgRNA) created by fusion of CRISPR RNA(crRNA) and trans-activating CRISPR RNA (tracrRNA) (Deng et al., 2017; Ferrara et al., 2019). Since CRISPR-Cas9 system relies on bacteria-derived Cas9 protein, codon optimization of the *cas9* gene is in most cases required for optimal expression of the system in eukaryotes (Nødvig et al., 2015; Zou et al., 2020). Moreover, it has been shown that constitutively expressed Cas9 can result in detrimental effects on some host genome structure in addition to resulting in unexpected phenotypic changes, such as delayed growth and loss of fitness (Enkler et al., 2016). Besides, off-target mutations and toxic effects on the cells associated with Cas9 overexpression have been reported (Jacobs et al., 2014; Foster et al., 2018).

Recently, an alternative approach, CRISPR/Cas9 RNP-mediated genome editing, has been adopted in numerous fungal studies. This approach entails *in vitro* assembly followed by the delivery of ribonucleoprotein complex consisting of the Cas9 protein and the gRNA to the cell. This method has a number of advantages, chief among them, the elimination of the need to find appropriate promoters for expressing the Cas9 protein and gRNAs (Abdallah et al., 2017; Ouedraogo and Tsang, 2020). Furthermore, CRISPR/Cas9 RNP-mediated genome editing has been shown to reduce the rate of off-target effects (Foster et al., 2018; Modrzejewski et al., 2020). Moreover, this approach allows for *in vitro* assessment of the efficiency of designed gRNAs to cleave the target region before their use in the transformation experiment (Abdallah et al., 2017; Ouedraogo and Tsang, 2020). CRISPR/Cas9 RNP-mediated genome editing has recently been applied successfully in filamentous fungi such as, *Aspergillus fumigatus* (Abdallah et al., 2017), *Magnaporthe oryzae* (Foster et al., 2018), *Aspergillus niger* (Kuivanen et al., 2019)*, Fusarium proliferatum* (Ferrara et al., 2019), *Trichoderma reesei* and *Cordyceps militaris* (Zou et al., 2020), and *Penicillium polonicum* (Valente et al., 2021).

Our previous study on discovery of bioactive secondary metabolites from fungal endophytes resulted in the isolation of palmarumycin compounds from *Lophiotrema* sp. F6932 (Gakuubi et al., 2022). Among the isolated compounds, palmarumycin C_8_ exhibited moderate antibacterial activity against *Staphylococcus aureus* presenting IC_90_ value of 18 μg/mL (Gakuubi et al., 2022). In the current work, we describe the development of a CRISPR/Cas9 RNP-mediated genome editing system that has enabled us to characterize a type 1 iterative PKS (PAL) that is responsible for palmarumycin and melanin biosynthesis in fungus.

## Materials and methods

### Fungal strain

*Lophiotrema* sp. F6932 was isolated from white mangrove also known as the Indian mangrove (*Avicennia officinalis*) in Pulau Ubin Island, Singapore, and stored at A*STAR’s Natural Product Library (NPL), Singapore Institute of Food and Biotechnology Innovation (SIFBI). Molecular identification of the fungal isolate was previously performed through amplification of the internal transcribed spacer 2 (ITS2) region of the rDNA gene using primer set ITS86F/ITS4 (White et al., 1990; Turenne et al., 1999). The ITS sequence generated was submitted to BLASTn program^1^ for the analysis of sequences similarity and the strain showed a match score of 99% with *Lophiotrema* sp. (MK587671.1). The ITS2 sequence for this strain is available in the GenBank database under the accession number OM791904. In the current work, an attempt was made to further establish the identity of *Lophiotrema* sp. F6932 and its phylogenetic relationships with closely related species. Multilocus sequence analysis (MLSA) was performed using five molecular markers, namely, three nuclear ribosomal genes, i.e., 18S nuclear small subunit ribosomal DNA (nrSSU), nuclear large subunit (nrLSU), and the entire internal transcribed spacer region (ITS), and two protein-coding loci, i.e., RNA polymerase II second largest subunit (RPB2) and translation elongation factor 1-alpha (*TEF1-α*). The sequences for the five molecular markers were obtained from *Lophiotrema* sp. F6932 genome data while those of closest relatives were retrieved from the NCBI database and recent references (Hashimoto et al., 2017; Andreasen et al., 2021). DNA gene sequences were aligned using the ClustalW algorithm in MEGA 7 software (Kumar et al., 2016). For each of the five loci, sequences were aligned individually and then concatenated for phylogenetic analyses based on Maximum Likelihood (ML) and Neighbor-Joining (NJ) methods.

### Whole-genome sequencing and bioinformatics analyses

Whole-genome sequencing of *Lophiotrema* sp. F6932 was done by Macrogen (South Korea) using a combination of PacBio Single Molecule, Real-Time (SMRT) Sequencing, and Illumina platforms followed by *de* novo assembly using bioinformatics software FALCON for PacBio long-reads and assembly polishing with Arrow. Illumina reads were applied for accurate genome sequence using Pilon for error correction*. Lophiotrema* sp. F6932 was found to have a 51.4% GC content with 37.1 Mb spread over 17 contigs. The genome assembly data was submitted to antibiotics & Secondary Metabolite Analysis Shell (antiSMASH) server for biosynthetic gene clusters (BGCs) prediction (Medema et al., 2011). *Lophiotrema* sp. F6932 was predicted to contain 14 T1PKS, 8 NRPS-like, 6 NRPS, and 6 terpene gene clusters in addition to a single T3PKS and NRPS-terpene hybrid cluster. Majority of the predicted biosynthetic gene clusters in *Lophiotrema* sp. F6932 were found to bear no similarity with any known gene clusters (Supplementary Table S1; Supplementary Figure S1). Analysis of antiSMASH data resulted in the identification of a putative 6.48 kb type I PKS that was predicted to be involved in the biosynthesis of palmarumycins in *Lophiotrema* sp. F6932. The sequence of palmarumycin (*PAL*) gene cluster was submitted to GenBank (Accession number ON768617). Moreover, *PAL* gene cluster showed a 100% similarity to the melanin biosynthetic gene cluster from phytopathogenic fungus *Bipolaris oryzae* based on antiSMASH analysis results (Moriwaki et al., 2004).

### Antibiotic sensitivity test

In order to assess the suitability of using hygromycin B phosphotransferase (*hph*) resistance gene as a selectable marker for knockout mutants, the sensitivity of *Lophiotrema* sp. F6932 to hygromycin B was evaluated by growing the wild-type strain in potato dextrose agar (PDA) plates supplemented with different concentrations of hygromycin B (InvivoGen, US). Three agar plugs (3 mm in diameter) were cut from the periphery of an actively growing 14-day old cultures of *Lophiotrema* sp. F6932 and grown in PDA plates containing 5, 10, 15, 20, and 50 μg/mL of hygromycin B. After 14 days of incubation, the concentration of the antibiotic that resulted in 100% growth inhibition was assessed visually.

### Protoplast isolation

A two-week-old plate of *Lophiotrema* sp. F6932 was flooded with 5 mL of sterile water and scraped gently using a cell spreader. The dislodged conidia were filtered through a layer of miracloth and 500 μL of conidia inoculated into 50 mL of PDB in a 250 mL flask and incubated at 24°C and 150 rpm for 72 h to allow the conidia to germinate. Mycelia were collected by filtration using miracloth and washed with 4 volumes of TPC buffer (50 mM potassium phosphate, pH 7.0, 0.8 M NaCl, and 20 mM MgSO_4_). The harvested mycelia were aseptically transferred to a 250 mL Erlenmeyer flask containing 100 mL of TPC buffer supplemented with 10 mM dithiothreitol (DTT) and incubated for 1 h at 30°C and 200 rpm. The mycelium was collected by filtration using miracloth and resuspended in 30 mL of protoplasting solution in a 250 mL flask and incubated at 30°C and 90 rpm. The protoplasting solution consisting of 1.2 M KCl and 20 mg/mL of cell wall-digesting enzyme was prepared an hour prior to protoplast isolation and had been stirred for 30 min followed by filtration through a 0.45 μm filter. The release of protoplasts was checked from 10 μL aliquots taken hourly.

Three commercial cell wall-digesting enzymes: Lysing enzymes from *Trichoderma harzianum* (Sigma-Aldrich, USA), yatalase (Takara Bio Inc., Japan), and snailase (Sigma-Aldrich, USA) were evaluated for their efficacy in protoplast generation with two of the best performing enzymes additionally tested in a cocktail consisting of 10 mg/mL of each enzyme. Protoplast yield was determined as the total number of protoplasts generated in the protoplasting solution at the end of the incubation period divided by the fresh weight of the mycelia used (i.e., protoplasts/g FW). After the incubation period and when most of the mycelia had been digested and maximum concentration of protoplast was recovered, the generated protoplasts were filtered first using 100 μm cell strainer followed by 20 μm cell strainer to remove residual mycelia. The solution containing the protoplast was centrifuged at 1000 × g for 5 min at 4°C. The protoplasts were gently resuspended in 10 mL chilled STC buffer (10 mM Tris–HCl; pH 7.5, 1 M sorbitol, 20 mM CaCl_2_) using a wide orifice pipet tip and centrifuged at 1000 × g for 5 min at 4°C. This centrifugation and resuspension step was repeated twice with a known volume of STC buffer used in the last resuspension step. Protoplast concentration was determined using a hemocytometer and the concentration was adjusted to a range of 1–5 × 10^8^ protoplasts/mL followed by the addition of 7% dimethyl sulfoxide (DMSO). Aliquots of 200 μL in 1.5 mL Eppendorf tubes were stored at –80°C until when required for the transformation process.

### Design of sgRNA and amplification of the HygR repair template

**Figure 1:**
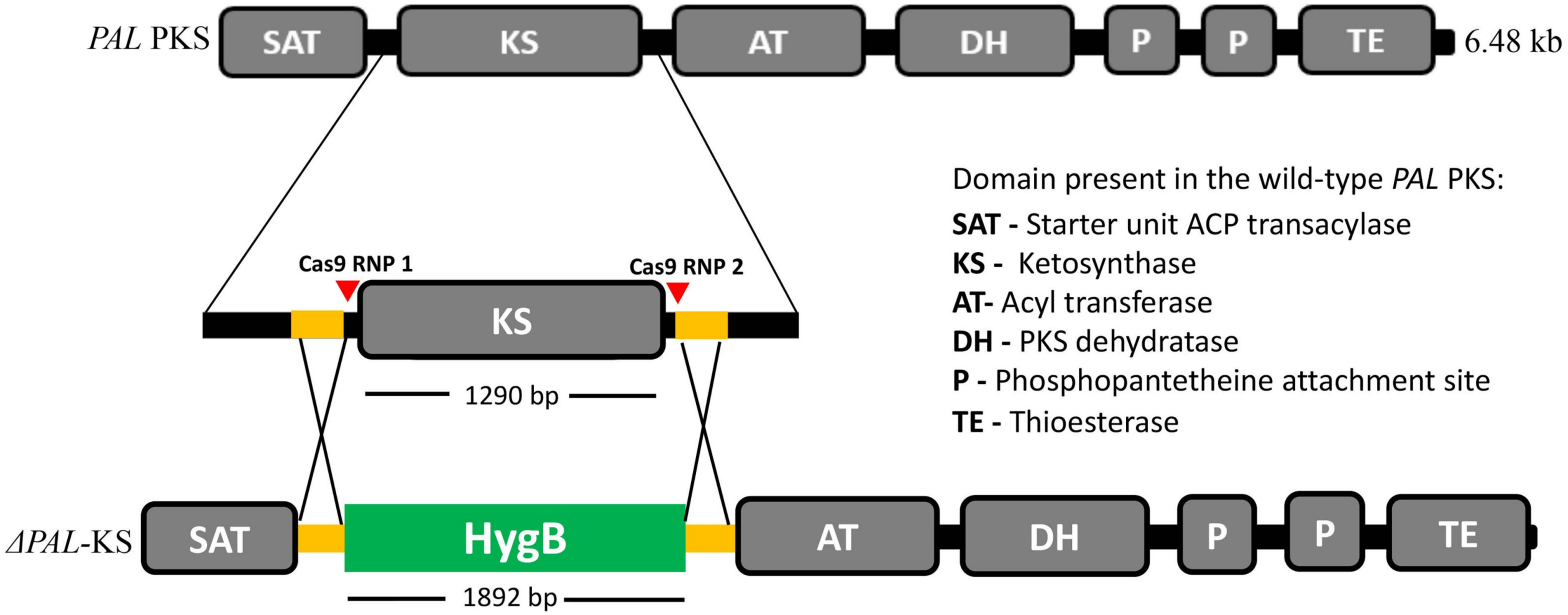
Schematic illustration of *PAL* KS domain deletion strategy by *in vitro*-assembled dual Cas-9 ribonucleoprotein coupled with homology-directed repair. The red triangles indicate the cleavage sites of the two Cas9-RNPs, while the orange and green segments indicate the 50-bp microhomology regions and the hygromycin B resistance cassette, respectively.

The KS domain of the putative palmarumycin *PAL* gene cluster is 1,290 bp long (Figure 1). The sgRNAs for KS domain deletion were designed using PhytoCRISP-Ex, a stand-alone program for the identification of target sequences for CRISPR-Cas9 editing, which was also used to check for potential off-targets (Rastogi et al., 2016). The design of the sgRNAs was based on the following parameters: (i) Two sgRNA cleavage sites located approximately 100 bp upstream and downstream of the KS domain; (ii) the protospacer adjacent motif (PAM) sequence for the sgRNAs was 5′-NGG-3′ (‘N’ being any nucleotide base). Hygromycin B phosphotransferase (*hph*) gene under the control of *A. nidulans trpC* promoter and terminator which had been previously cloned into the vector pUC19 in the *E. coli* cells was used as a selectable marker. Plasmid DNA was isolated from *E. coli* harboring the plasmid of interest using QIAprep Spin Miniprep kit (Qiagen, Germany) following the manufacturers’ instructions. For generation of the repair template for CRISPR/Cas9 RNP-mediated genome editing, hygromycin B phosphotransferase expression cassette was PCR-amplified using primer set 50-bp-HomoF/50-bp-HomoR (Table 1). The resultant PCR fragments were purified using MEGAquick-spin total fragment DNA purification kit (iNtRON Biotechnology, South Korea). The purified PCR products were sequenced to check for any error and then used as the repair template consisting of 1892 bp hygromycin B resistance cassette flanked on both sides by a 50 bp microhomology arm targeting the two RNP cleavage sites (Figure 1).

**Table 1:** Primers and oligonucleotides used in this study.

Name	Sequence (5′-3′)	Type or purpose
gRNA1	GTAACGGTTTGCATCGACCT-GGG	5’ crRNA (Sequence – PAM)
gRNA2	GAGTCCACACGTTGTGATCG-CGG	3’ crRNA (Sequence – PAM)
50-bp-HomoF	AACAGCCTGGCATCGGCCCTAAAGGCAGGTGGCCAGACCTCGATCTCTGTgctagtggaggtcaacac	Amplification of HDR-HygR repair template with 50-bp microhomology arm
50-bp-HomoR	CTTCAACGAAGCAATAGACCTGGCAGTAACCGTAACGGTTTGCATCGACCaacccaggggctggtgac	Amplification of HDR-HygR repair template with 50-bp microhomology arm
ChkF	TTCAGCAACAGCGAGTGC	Verification of knockout mutants, and amplification of template for *in vitro* Cas9 cleavage assay to check for gRNA efficiency
ChkR	GAGACACAGACAGCGACG	Verification of knockout mutants, and amplification of template for *in vitro* Cas9 cleavage assay to check for gRNA efficiency
KSF1	AGGAGAGCTGGTCCACCATC	Verification of knockout mutants
KSR1	GTGCTAACGTCAGTGCCATC	Verification of knockout mutants
HyF1	TTTCGGCTCCAACAATGTCC	Verification of knockout mutants (Hygromycin resistance cassette)
HyR1	GCTGCTCCATACAAGCCAAC	Verification of knockout mutants (Hygromycin resistance cassette)
ITS86F	GTGAATCATCGAATCTTTGAA	Amplification of the ITS2 region in rDNA gene
ITS4	TCCTCCGCTTATTGATATGC	Amplification of the ITS2 region in rDNA gene

Microhomology arms used for homology-directed repair are underlined while sequences specific for amplification of HygR cassette are in lowercase letters.

### Assessment of sgRNA efficiency by *in vitro* DNA cleavage assay

Prior to the use of the designed sgRNA for CRISPR-Cas9 mediated gene editing, it is important to test their efficiency in cleaving the target DNA. *In vitro* Cas9 cleavage assay entails three steps, namely, (a) PCR-amplification of a target DNA template containing one or several sgRNA target cleavage sites; (b) *in vitro* cleavage of the target sequence by recombinant Cas9 and candidate sgRNA; (c) separation of cleavage products by agarose gel electrophoresis. The procedure described by (Mehravar et al., 2019; Bente et al., 2020) with some modifications were followed for *in vitro* pre-validation of the designed sgRNAs. *Lophiotrema* sp. F6932 genomic DNA was isolated from mycelia harvested from fleshy subcultured malt extract agar (MEA) plates. Approximately 100 mg of mycelia were scraped using sterile toothpick and grounded to powder in liquid nitrogen using a sterile mortar and pestle. DNA was extracted using DNeasy PowerSoil Kit (Qiagen, Germany) following the manufacturers’ instructions. Cleavage activity was assessed on a 1,664-bp PCR product encompassing the *PAL* KS domain amplified from the genomic DNA using primer set ChkF/ChkR (Table 1). Duplex RNA (crRNA and tracrRNA) were resuspended in nuclease-free duplex buffer (IDT, United States) to a stock solution of 100 μM. The two were then mixed in equimolar concentration to a final concentration of 10 μM using nuclease-free duplex buffer, annealed by heating at 95°C for 5 min in a thermomixer followed by cooling to room temperature for 15 min. To assemble the CRISPR RNP complex, crRNA:tracrRNA duplex was combined with Alt-R *S. pyogenes* Cas9 nuclease (IDT, Singapore) in equimolar amounts and incubated at room temperature for 10 min. The cleavage reaction was performed by mixing the following: 1 μL of 10 × Cas9 nuclease reaction buffer (200 mM HEPES, 1 M NaCl, 50 mM MgCl_2_, 10 mM EDTA, pH 6.5), 1 μL of 1 μM Cas9 RNP, 3 μL of 10 nM of target DNA template and 5 μL of nuclease-free water. The mixtures were incubated at 37°C for 1 h. After incubation, 1 μL of proteinase K (20 mg/mL) was added to the reaction followed by incubation of the mixture at 56°C for 10 min to release the DNA from the Cas9 endonuclease. The products of each reaction were then visualized by electrophoresis on 1% agarose gel stained with SYBR safe DNA gel stain. All the oligonucleotides used in the current study were purchased from Integrated DNA Technologies (IDT, Singapore) (Table 1).

### PEG-mediated protoplast fungal transformation

PEG-mediated protoplast transformation was performed following the procedure described in the literature with some modifications (Abdallah et al., 2017). The two gRNAs were generated by separately hybridizing each crRNA to the tracrRNA using nuclease-free duplex buffer to a final concentration of 33 μM. The mixtures were heated at 95°C for 5 min in a thermomixer followed by cooling to room temperature for 15 min. To generate the Cas9-NRP complexes, 1.5 μL of each gRNA was separately mixed with 0.75 μL of 1 μg/μL Cas9 enzyme and 11 μL of 1× PBS buffer at room temperature and the mixtures were incubated at room temperature for 10 min. For the control, Cas9 enzyme was replaced with PBS. The two reaction mixtures were then combined to a final volume of 26.5 μL at room temperature and used for protoplast transformation. Protoplasts stored at −80°C were thawed on ice and centrifuged at 3000 × *g* for 5 min at 4°C to remove DMSO. The protoplasts were resuspended in 400 μL of chilled STC buffer, centrifuged, and finally resuspended in 200 μL STC buffer before transferring into a sterile 15 mL conical tube containing 26.5 μL Cas9-NRP complex. This was followed by the addition of 5 μg of purified repair template and 25 μL of PEG-CaCl_2_ buffer (30% [wt/vol] PEG 3350, 1 M CaCl_2_, 50 mM Tris–HCl, pH 7.5), then incubation on ice for 90 min. Subsequently, 1.25 mL of PEG-CaCl_2_ buffer was added followed by incubation at room temperature for 20 min. The mixture was diluted to a total volume of 3 mL with STC buffer and transferred into 7 mL of warm molten MEA (1.5% wt/vol agar) supplemented with 1.2 M sorbitol in a 50 mL conical tube. The mixture was swirled gently and poured into Petri dish, allowed to cool, and incubated at 24°C for 72 h. Subsequently, the plates were overlaid with 10 mL molten MEA (1.5% wt/vol agar) supplemented with 1.2 M sorbitol and 50 μg/mL of hygromycin B. The negative control plate (lacking Cas9 RNP) was overlaid with MEA without the antibiotics while the positive control plate (with the Cas9 RNP) had MEA containing the antibiotics. The plates were incubated at 24°C for four additional days with the growth of colonies on the plates monitored regularly. Single colonies growing in the experimental plates were subcultured on fresh MEA plates containing 50 μg/mL of hygromycin B.

### Screening for stable transformants

Putative deletion mutants that grew through the overlay MEA supplemented with hygromycin B were transferred to new MEA plates containing the antibiotic. After 10 days of growth, the mutants were subcultured into new MEA plates containing the selection antibiotics. Mycelia were cut from the periphery of an actively growing 10-day old cultures of these plates and subcultured in non-selective media (MEA without hygromycin B) and incubated for 10 days. This step was repeated for three successive times. Finally, putative mutants growing on non-selective medium were subcultured back to selective media to confirm their resistance toward hygromycin B. Growth of the mutants in MEA plates supplemented with 50 μg/mL of hygromycin B at this stage indicated that they were mitotically stable. Successful deletion of the KS domain and integration of hygromycin B resistance gene *hph* into the transformants genome was confirmed by PCR and sequencing.

### Fungal cultures extraction and liquid chromatography-mass spectrometry analysis

The wild-type strain and the two arbitrarily selected *Lophiotrema* sp. F6932 *ΔPAL* KS mutants were fermented in CF02LB media for the generation of crude extracts. The fungal strains were grown in 50 mL media in 250 mL conical flasks by inoculating three mycelial discs (5 mm in diameter) into each of the media flasks. The cultures were incubated for 14 days in a shaking incubator at 24°C and 200 rpm. The cultures were then frozen at −80°C and freeze-dried in a vacuum freeze-dryer to expel all moisture before extracted using MeOH. The crude extracts were dissolved in 1:1 ratio of DMSO and 50% MeCN/H_2_O (0.1% formic acid) to make solutions at 20 mg/mL concentration. After centrifugation at 14,600 rpm for 5 min, the supernatants were transferred to LC vials and a volume of 2 μL of the extract was subjected to LC-HRESIMS. LC-HRESIMS was performed on an Agilent UHPLC 1290 Infinity coupled to Agilent 6,540 accurate-mass quadrupole time-of-flight (QTOF) mass spectrometer equipped with a splitter and ESI source. Liquid chromatography was carried out using an Agilent Poroshell 120 SB-C18 (4.6 × 75 mm, 2.7 μm) using MeCN and H_2_O, both containing 0.1% formic acid, as mobile phase. Initially, an isocratic system of 5% of MeCN/H_2_O was employed for 2 min, followed by a linear gradient to 100% MeCN over 16 min and an isocratic wash of 100% MeCN for 5 min, with the flow rate set at 2 mL/min. Mass spectrometer parameters were set as previously described (Sirota et al., 2018). MS data were analyzed and processed using Agilent MassHunter Qualitative Analysis Version 10.0. The presence of palmarumycins was confirmed by comparison with our in-house standard compounds library (Gakuubi et al., 2022). All solvents for extraction and LC–MS were of chromatographic grade.

## Results

### Characterization of *Lophiotrema* sp. F6932 by phylogenetic analysis

*Lophiotrema* sp. F6932 was previously identified on the basis of sequencing analysis of ITS2 region of the rDNA gene. To further establish the identity of this isolate and its phylogeny, multilocus sequence analysis was conducted using an aligned sequence dataset comprising of 1,014 nucleotide positions from nrSSU, 256 from ITS, 1116 from nrLSU, 1,004 from RPB2, and 899 from *TEF1-α*. Phylogenetic analysis was performed using a data set of 21 taxa representing 9 genera in the family *Lophiotremataceae* with *Crassiparies quadrisporus* HHUF 30409 as the outgroup. The resultant ML and NJ phylogenetic tree analyses of the combined datasets yielded the best scoring trees for *Lophiotremataceae* (Supplementary Figures S2, S3) and show that F6932 is close to *L. eburnoides* HHUF 30079, with 91.97% identity score in the ITS locus. Based on MLSA analysis of the five loci, *Lophiotrema* sp. F6932 differs from *L. eburnoides* HHUF 30079 by 20 nucleotides (nt) in the D1/D2 domains in the ITS region. Variations are also observed in other gene sequences such as LSU (11 nt), nrSSU (3 nt), *TEF1-α* (40 nt), and RPB2 (137 nt) when compared to *L. eburnoides* HHUF 30079. These results indicate that *Lophiotrema* sp. F6932 represents a potentially novel species within the Genus *Lophiotrema.* The sequences for the nrSSU, nrLSU, ITS, RPB2, and *TEF1-α* for this strain are available in the GenBank database and their accession numbers are given in Supplementary Table S2. The sensitivity of *Lophiotrema* sp. F6932 to hygromycin B was evaluated by growing the wild-type strain in plates supplemented with different concentrations of the antibiotic. PDA plates supplemented with 20 μg/mL of hygromycin B resulted in complete growth inhibition of *Lophiotrema* sp. F6932 wild-type strain, an indication that hygromycin B resistance gene would be suited for used as a selectable marker for *Lophiotrema* sp. F6932 knockouts mutants (Supplementary Figure S4).

### Protoplast isolation from *Lophiotrema* sp. F6932

**Figure 2:**
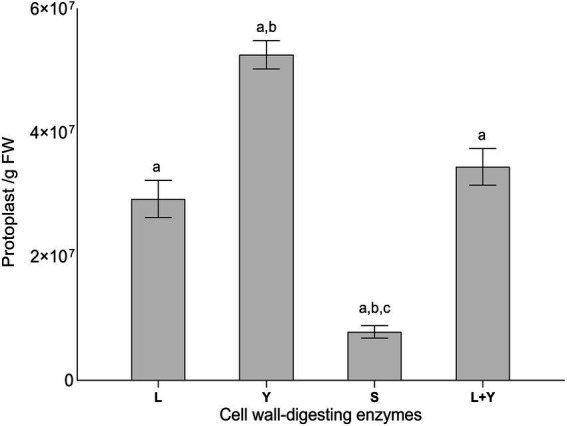
Protoplasts yield from *Lophiotrema* sp. F6932 using different cell wall-digesting enzymes (L = lysing enzymes from *T. harzianum*, Y = yatalase, S = snailase, and L + Y = cocktail of L and Y). Results are expressed as means ± SEM from three biological replicates. Different lowercase letters (a–c) among the treatments indicate statistically significant differences between means by one-way ANOVA with Tukey–Kramer multiple comparison test (*p* < 0.05).

No protocol exists in the literature for protoplast isolation from members of *Lophiotrema* genus or even the closely related genus *Lophiostoma*. Thus, we used several existing protoplast isolation methods as the basis for developing a simple yet efficient method for protoplast generation from *Lophiotrema* sp. F6932 (Abdallah et al., 2017; Roth and Chilvers, 2019; Ning et al., 2022). Preliminary studies had shown that potato dextrose broth (PDB) was the best media for generating mycelia for protoplast isolation from this fungus compared with two other media; malt extract broth and Sabouraud dextrose broth (Supplementary Figure S5). Three enzymes namely; lysing enzymes from *T. harzianum*, yatalase, and snailase at the concentration of 20 mg/mL, were tested for their efficacy in protoplast generation from *Lophiotrema* sp. F6932. After 5 h incubation, most of the mycelia was digested with maximum concentration of protoplast recovered. The best performing enzyme was yatalase with a mean yield of 5.2 × 10^7^ protoplasts/g fresh weight (FW), followed by the lysing enzymes from *T. harzianum* with a mean yield of 2.9 × 10^7^ protoplasts/g FW. The snailase enzyme had the lowest protoplast generation capacity with a mean yield of 7.8 × 10^6^ protoplasts/g FW. Protoplast yield by yatalase enzyme was significantly higher than that obtained with either lysing enzymes from *T. harzianum* or a cocktail of lysing enzymes from *T. harzianum* and yatalase. There were, however, no significant differences in the yield of protoplasts between lysing enzymes from *T. harzianum* and the cocktail of the two enzymes (Figure 2).

### *In vitro* cleavage efficiency of sgRNAs

The two sgRNAs (Table 1) that were designed for use in this study were subjected to *in vitro* cleavage assay to assess their ability to cleave the target 1,664 bp PCR fragment encompassing the *PAL* KS domain. Analysis of the cleavage reaction using agarose gel electrophoresis resulted in the expected band sizes (Supplementary Figure S6). Cleavage using gRNA1 produced expected fragments of approximately 1,494 bp and 170 bp, gRNA2 produced bands of approximately 1,557 bp and 107 bp while the use of the two gRNAs together resulted in expected bands of 1,387 bp, 107 bp, and 170 bp (Supplementary Figure S6). On the basis of the *in vitro* cleavage assay results, the two gRNA were deemed to be efficient in the cleavage of the target sites during the polyethylene glycol (PEG)-mediated transformation experiment.

### Morphological characteristics of RNP-mediated CRISPR-Cas9 deletion mutants

**Figure 3:**
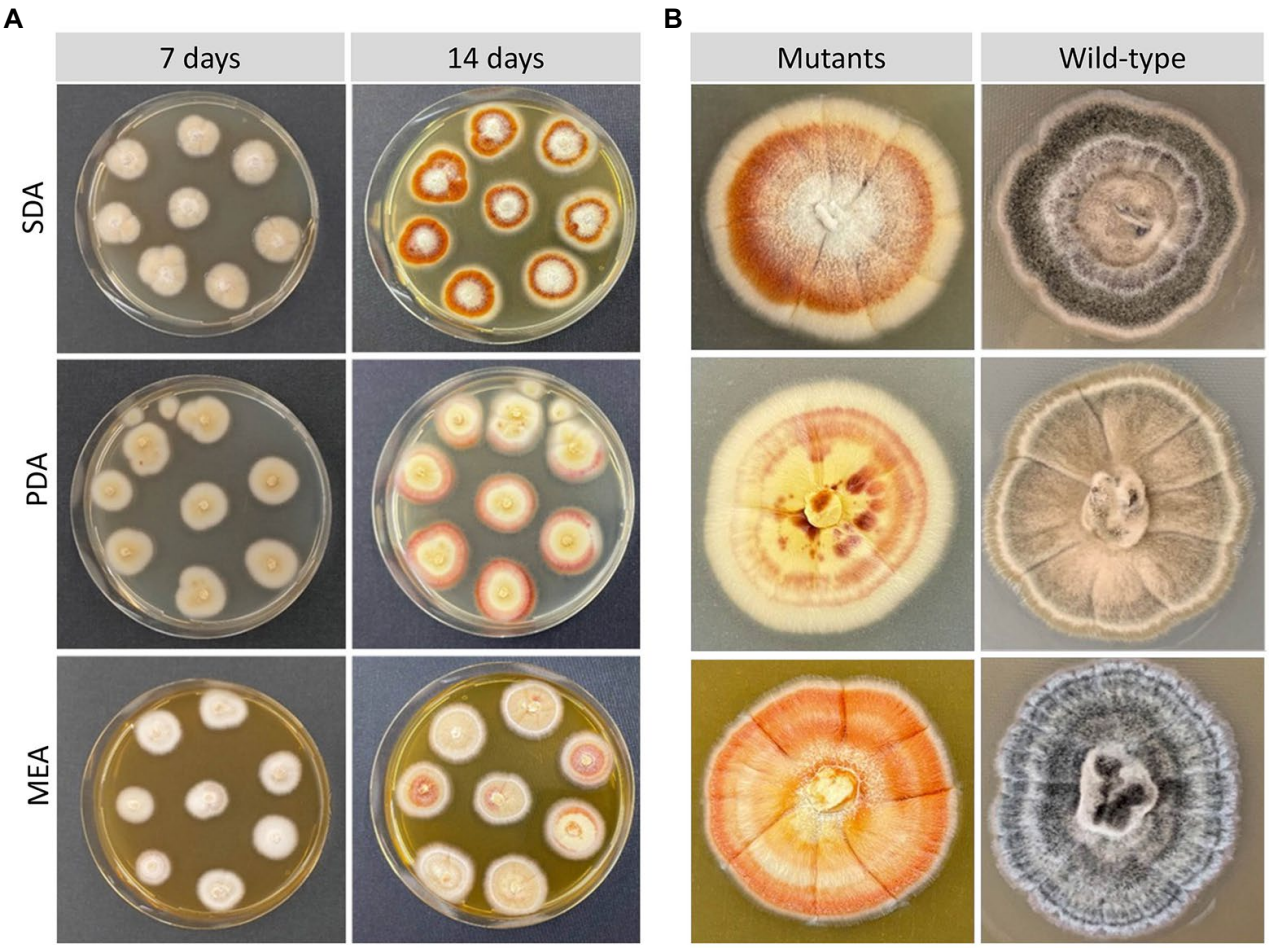
Morphological characteristics of *Lophiotrema* sp. F6932 CRISPR/Cas9-induced mutants. **(A)** Morphology of the deletion mutants after 7 and 14 days of growth in SDA, PDA, and MEA media; **(B)** close-up view comparison of the colony morphology of the deletion mutants and the wild-type strain after 21 days of growth in SDB, PDA, and MEA media.

The morphology of *Lophiotrema* sp. F6932 *ΔPAL* KS mutants was evaluated after 7 and 14 days of growth on three common media; malt extract agar (MEA), potato dextrose agar (PDA) and Sabouraud dextrose agar (SDA). All the deletion mutants displayed completely different morphological characteristics from the wild-type strain. In all the three tested media, the mutants had a white to creamy appearance in contrast to characteristic black color of the wild-type strain after 7 days (Figure 3A). However, by the 14^th^ day of incubation, the mutants’ pigmentation changed in all the three media. In SDA, the colonies showed three distinct regions; creamy white peripheries, surrounding an orange-red middle section, and a creamy white patch at the center. In PDA, the colonies had orange-red peripheries surrounding a creamy white/yellowish in the middle, while in MEA, the mutants exhibited mixed colony morphologies with some colonies retaining the creamy white appearance, while others had, in addition, some orange-red patches (Figure 3A). When the mutants were grown beyond 3 weeks, the mutants retained the three distinct shades of color in SDA, while in PDA, the mutants retained the creamy white peripheries with concentric rings of orange-red in the middle and yellowish and orange-red patches at the center of the colonies (Figure 3B). In MEA, the orange-red color spread to the entire colony (Figure 3B).

**Figure 4:**
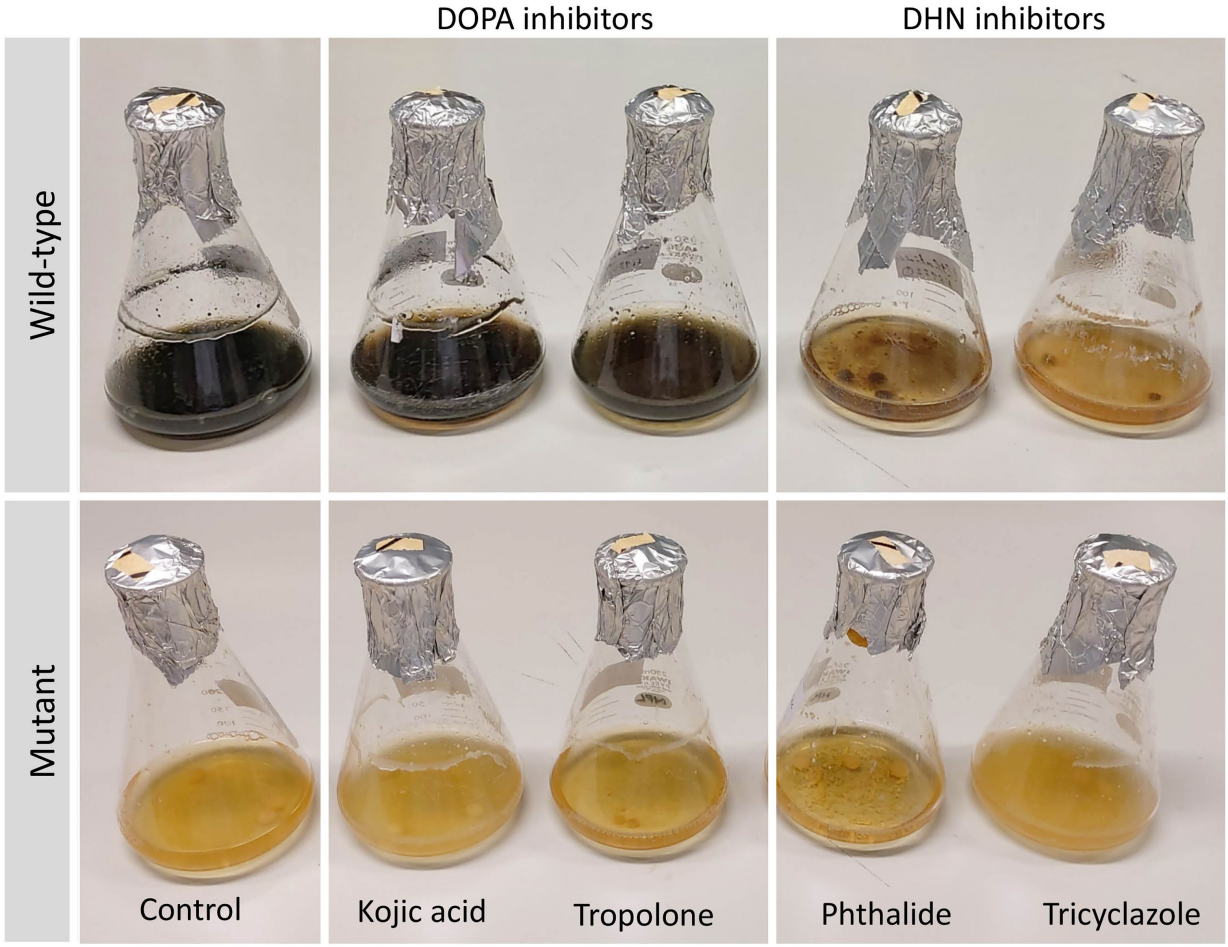
Growth of *Lophiotrema* sp. F6932 wild-type strain and CRISPR/Cas9-induced mutants in PDB in the presence of different melanin synthesis inhibitors after 6 days. The concentration of kojic acid, phthalide, and tricyclazole used was 100 μg/mL while 12.5 μg/mL of tropolone was used. The results show that melanin biosynthesis in wild-type strain was inhibited only in the presence of the two DHN melanin inhibitors, phthalide and tricyclazole, suggesting that this strain produces melanin through the 1,8-dihydroxynaphthalene (DHN) pathway.

In addition to the observed differences in colony morphologies between the deletion mutants and the wild-type strain, the deletion mutants were moreover able to grow in the selective media even after the concentration of hygromycin B was increased four-fold of what was used in the transformation experiment (200 μg/mL). There were furthermore clear differences in the color of mutant-derived crude extracts when compared with those from the wild-type strain (Supplementary Figure S7). Growth of *Lophiotrema* sp. F6932 wild-type strain in the presence of 1,8-dihydroxynaphthalene (DHN) and 3,4-dihydroxyphenylalanine (DOPA) melanin synthesis inhibitors showed the loss of melanin production in cultures grown in the presence of the two DHN melanin synthesis inhibitors, phthalide and tricyclazole (Figure 4). This confirms that *Lophiotrema* sp. F6932 similar to *B. oryzae,* produces melanin through the DHN pathway. Cultures of the mutants grown in PDB lacked the characteristic dark color of the wild-type strain, suggesting that they were unable to produce melanin. Furthermore, both DHN and DOPA melanin synthesis inhibitors did not have any observable effects on the culture of the mutants (Figure 4).

### Molecular analysis of hygromycin-resistant mutants

**Figure 5:**
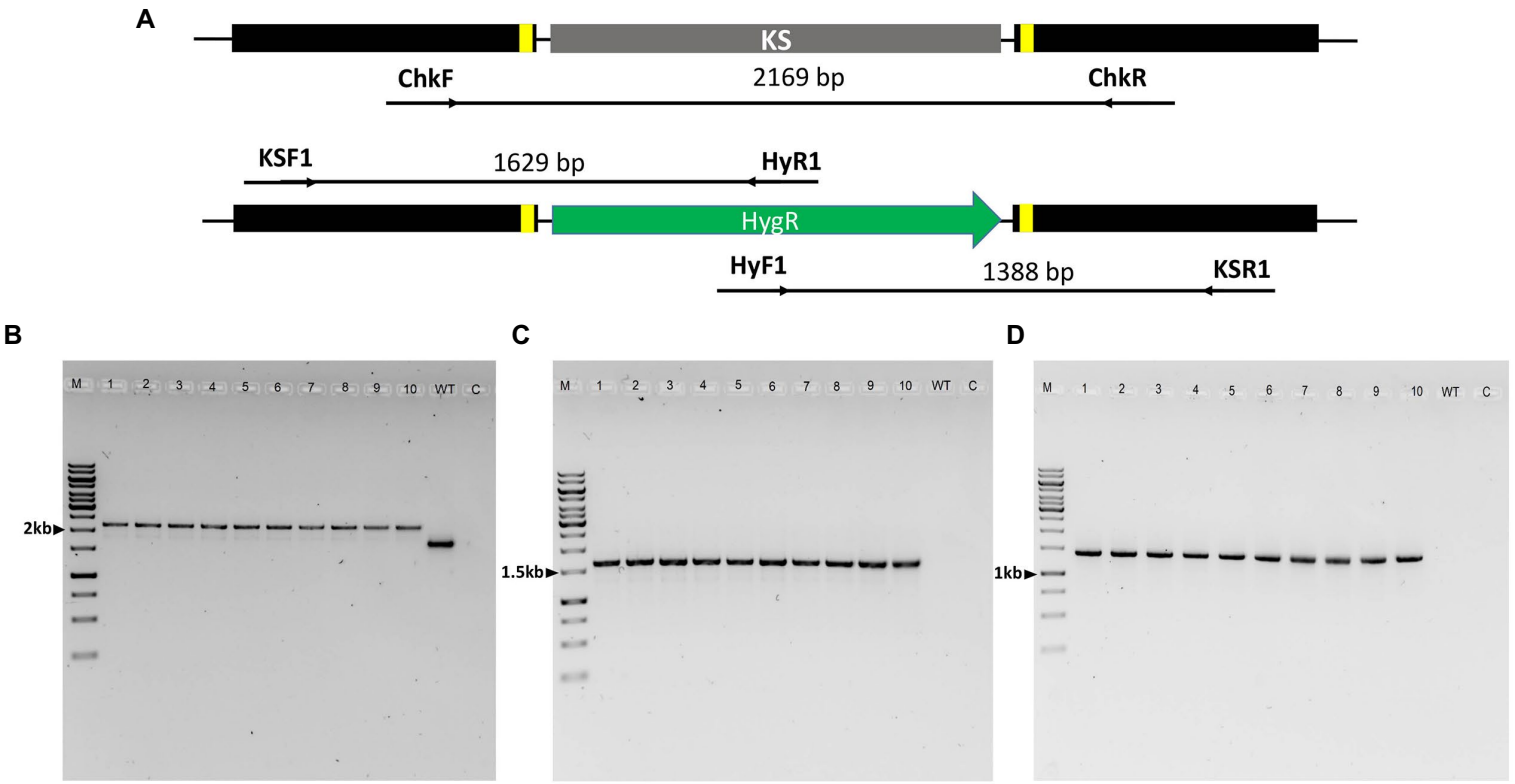
PCR analysis of *Lophiotrema* sp. F6932 Δ*PAL* KS deletion mutants. **(A)** An overview of the *PAL* KS domain locus in the wild-type strain and integration of HygR after CRISPR-Cas9 RNP-mediated deletion of the KS domain; **(B)** PCR amplification of HDR-HygR repair template integration locus spanning the cleavage sites of Cas9-RNPs complexes using ChkF/ChkR primer set showing the expected 2,169 bp and 1,664 bp bands in the mutants and wild-type strain, respectively; **(C)** PCR amplification of the deletion mutants and wild-type strain using primer sets KSF1/HyR1 and **(D)** HyF1/KSR1. M *=* 1 kb DNA ladder, 1–10 = Δ*PAL* KS deletion mutants resistant to hygromycin B, WT = wild-type strain, and C = Control. The full-length gel pictures are shown in Supplementary Figures S8A–C.

The identity of the deletion mutants was confirmed through sequencing of the ITS2 region using primer set ITS86F/ITS4. The putative Δ*PAL* KS deletion mutants were then screened by PCR amplification and sequencing. PCR amplification of the genomic sequence upstream and downstream of the two Cas9-RNPs cleavage sites was performed from genomic DNA isolated from ten randomly selected mutants using ChkF/ChkR primer set (Figure 5A). This resulted in the expected fragment of 2,169 bp consistent with the correct integration of the HygR cassette within the cleavage sites in all the 10 mutants, while a 1,664 bp band was obtained in the wild-type strain (Figure 5B). Analysis of the sequencing results of resultant PCR products revealed the presence of the entire HygR cassette sequence in all the 10 mutants. Furthermore, to confirm the orientation of the integrated HygR cassette, PCR amplification of the 10 deletion mutants and wild-type strain was performed using primer sets KSF1/HyR1 and HyF1/KSR1 resulting in the expected band of approximately 1,629 bp and 1,388 bp in all the 10 deletion mutants, respectively, (Figures 5C,D). As would be expected, no band was obtained in the wild-type strain since HyR1 and HyF1 primers are located within the HygR cassette (Figures 5C,D).

### Palmarumycin analysis results

**Figure 6:**
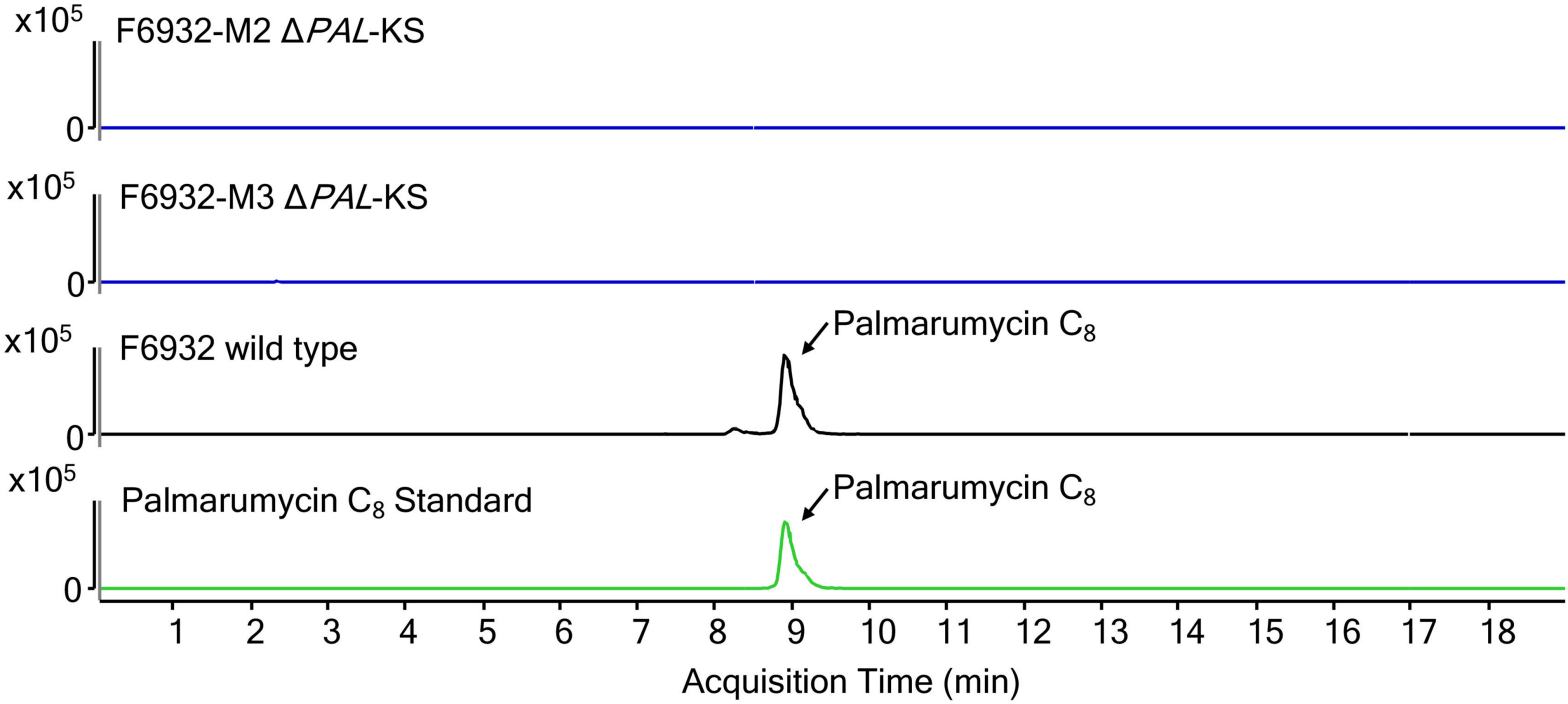
Extracted ion chromatograms (EIC) for palmarumycin C_8_ of cultures extracts from wild-type *Lophiotrema* sp. F6932 (black line) and two arbitrarily selected Δ*PAL*-KS mutants (blue lines). Palmarumycin C_8_ (*m/z* = 385.0473 ± 10 ppm, RT 8.8 min).

The target gene for this study was the polyketide synthase *PAL*, which was predicted to be involved in the biosynthesis of palmarumycins. We hypothesized that a knockout of the KS domain in *PAL* would disrupt the functioning of the gene and consequently the production of the palmarumycins. Therefore, LC–MS analysis was performed on MeOH extracts obtained from the cultures of *Lophiotrema* sp. F6932 wild-type strain and two randomly selected hygromycin B resistant mutants grown under the same conditions. The LC–MS analysis results confirmed the presence of palmarumycins in wild-type strain extracts but not in mutant-derived extracts (Figure 6; Supplementary Figure S9). These results thus confirm the involvement of palmarumycin synthase gene (*PAL*) in palmarumycins biosynthesis in *Lophiotrema* sp. F6932.

## Discussion

The emergence of CRISPR-Cas9 genetic editing technology has opened up new avenues for genetic manipulation of practically all type of fungal strains thus overcoming many of the challenges associated with the classical methods of genome engineering of these microorganisms (Nødvig et al., 2015; Schuster and Kahmann, 2019). In this study, we report the successful CRISPR/Cas9-based genome editing in *Lophiotrema* sp. F6932 using *in vitro* assembled Cas9-RNPs coupled with homology-directed repair. We used a similar approach employed in the transformation of *A. fumigatus* using *in vitro*-assembled Cas9-gRNA RNP coupled with microhomology repair template (Abdallah et al., 2017). This method does not require the generation of a plasmid harboring the Cas9 expression cassette but rather, entails the use of a complex of purified and commercially available Cas9 enzyme and sgRNAs to direct double-strand breaks (DSBs) upstream and downstream of the targeted region (Abdallah et al., 2017; Ferrara et al., 2019; Valente et al., 2021). This is followed by HDR using repair templates harboring homology arms on both sides. We successfully employed this approach in the validation of palmarumycin (*PAL*) biosynthetic gene cluster in *Lophiotrema* sp. F6932 through deletion of KS domain followed by homology-directed repair using hygromycin resistance cassette flanked by 50 bp of homology on both sides of the DSBs.

Among the many barriers toward genetic transformation of filamentous fungi is the difficulty encountered in generating high-quality protoplasts from some fungal strains (Roth and Chilvers, 2019). This is compounded by the fact that some fungal strains are not amenable to protoplasting (McCluskey and Baker, 2017). Different fungal strains possess cell walls with unique properties requiring the use of specific protoplast isolation conditions including the types and concentrations of cell wall-digesting enzymes (Li et al., 2017; Roth and Chilvers, 2019). Moreover, selection of an appropriate type and concentration of an osmotic stabilizer in the protoplasting medium is critical in preventing lysis or shriveling of protoplast due to osmotic stress (Ren et al., 2018; De Wu and Chou, 2019). Other than the enzymes and osmotic stabilizers, other factors such as enzymatic hydrolysis temperature, duration of incubation, fungal age and mycelium status need to be optimized for each strain (Jin et al., 2020; Ning et al., 2022). For example, depending on whether the fungal strain is slow or fast-growing, very short culture time may yield less mycelium and thus low yield of protoplast. On the other hand, prolonged culture time may result in aged mycelia whose thick cell wall may be resistant to enzymatic hydrolysis (Wei et al., 2012; Ning et al., 2022).

Because of the many differences among fungal strains, most often, the existing protoplast isolation protocols have to be optimized, and at times completely new protocols need to be developed for efficient protoplast isolation and eventual transformation, especially among the uncommon fungal strains (Li et al., 2017). *Lophiotrema* sp. F6932 is a slow-growing and heavly-melanized fungus; two characteristics that have been shown to contribute significantly to recalcitrant to protoplast generation and amenability to genetic manipulation in filamentous fungi (Ishikawa et al., 2010; Voigt et al., 2020). No protoplast isolation protocol exists in literature for fungi belonging to *Lophiotrema* genus or even the closely related phylogenetic group *Lophiostoma.* Our attempt to use reported protoplast isolation methods failed to generate the quantity of protoplasts needed for the transformation process for *Lophiotrema* sp. F6932. Therefore, through the optimization of various growth parameters and the used of different types and concentrations of commercial enzymes and osmotic stabilizers, and borrowing from existing protoplast isolation protocols (Abdallah et al., 2017; Roth and Chilvers, 2019; Ning et al., 2022), we designed a simple yet efficient method for protoplast isolation from *Lophiotrema* sp. F6932. Of all the factors that we optimized during the design of protoplast isolation protocol from this fungus, the effects of different commercial enzymes on protoplast yield were studied in detail. To this end, three enzymes; lysing enzymes from *T. harzianum*, yatalase, and snailase at the concentration of 20 mg/mL, were tested for their efficacy in protoplast generation. Two of the best performing enzymes were additionally tested in a cocktail consisting of 10 mg/mL of each enzyme. There were significant differences in protoplast yield among the three enzymes used. Yatalase resulted in significantly higher yield of protoplast compared with lysing enzymes from *T. harzianum* and snailase enzyme. Furthermore, a cocktail of yatalase and lysing enzymes from *T. harzianum,* two of the best performing enzymes produced significantly lower yields of protoplasts than yatalase used alone. Generally, different fungal strains will vary in their sensitivity to different enzymes because of the differences in the cell wall composition and structure (Jin et al., 2020). Therefore, depending on the nature of the fungus cell wall, different cell-wall digesting enzymes will act on different sites of the cell wall, resulting in variations in the level of cell wall hydrolysis and consequently the quantity of protoplasts released (Ning et al., 2022). Thus, the observed difference in the yield of protoplasts in the current study is indicative of differences in the sensitivity of *Lophiotrema* sp. F6932 to different enzymes due to the fungus cell wall composition.

A number of factors have been found to have an influence on the overall success rate of homologous recombination in PEG-mediated fungal transformation. For example, in one study, the amount of repair template, length of microhomology region, and concentration of Cas9 nuclease were found to have an influence on the efficiency of CRISPR-Cas9 mediated gene editing in wild-type and a *ΔakuB* strains of *A. fumigatus* (Abdallah et al., 2017). The study found that the use of 2 μg of repair template franked by either 35 bp or 50 bp homologous sequence resulted in high-efficiency gene deletion (up to 97%) in the *ΔakuB* strain. However, using the same concentration of the repair template flanked by 35 bp and 50 bp homologous sequences resulted in 46 and 74% gene deletion efficiency in the wild-type strain, respectively (Abdallah et al., 2017). In another study, a modified CRISPR/Cas9 gene editing system was developed for *A. niger* in which the efficiency of gene replacement was tested using repair templates with 100 bp and 500 bp arm lengths. Interestingly, the results revealed that using 100 bp homology arms was sufficient to obtain recombination at a high frequency (Zhang et al., 2019). In another study, a CRISPR-Cas9 RNP-mediated co-editing and counter selection system was developed for rice blast fungus *Magnaporthe oryzae* using 30 bp and 40 bp homologous sequences. The results demonstrated that 30 bp homologous sequence was sufficient in promoting homologous recombination of the repair template resulting in successful generation of *ALB1* mutants that were unable to produce melanin (Foster et al., 2018). In the current study, homologous recombination frequency of 71.4% was achieved using 50 bp homology arms and 5 μg of repair template. These results are quite comparable to those obtained in a study on RNP-directed genome editing of *Trichoderma reesei*, where homologous recombination frequency of 73.9% was obtained with 50 bp homology (Zou et al., 2020). It would be interesting to assess how variation of these among other factors would impact on the overall success rate of homologous recombination in the transformation of *Lophiotrema* sp. F6932 in the future.

**Figure 7:**
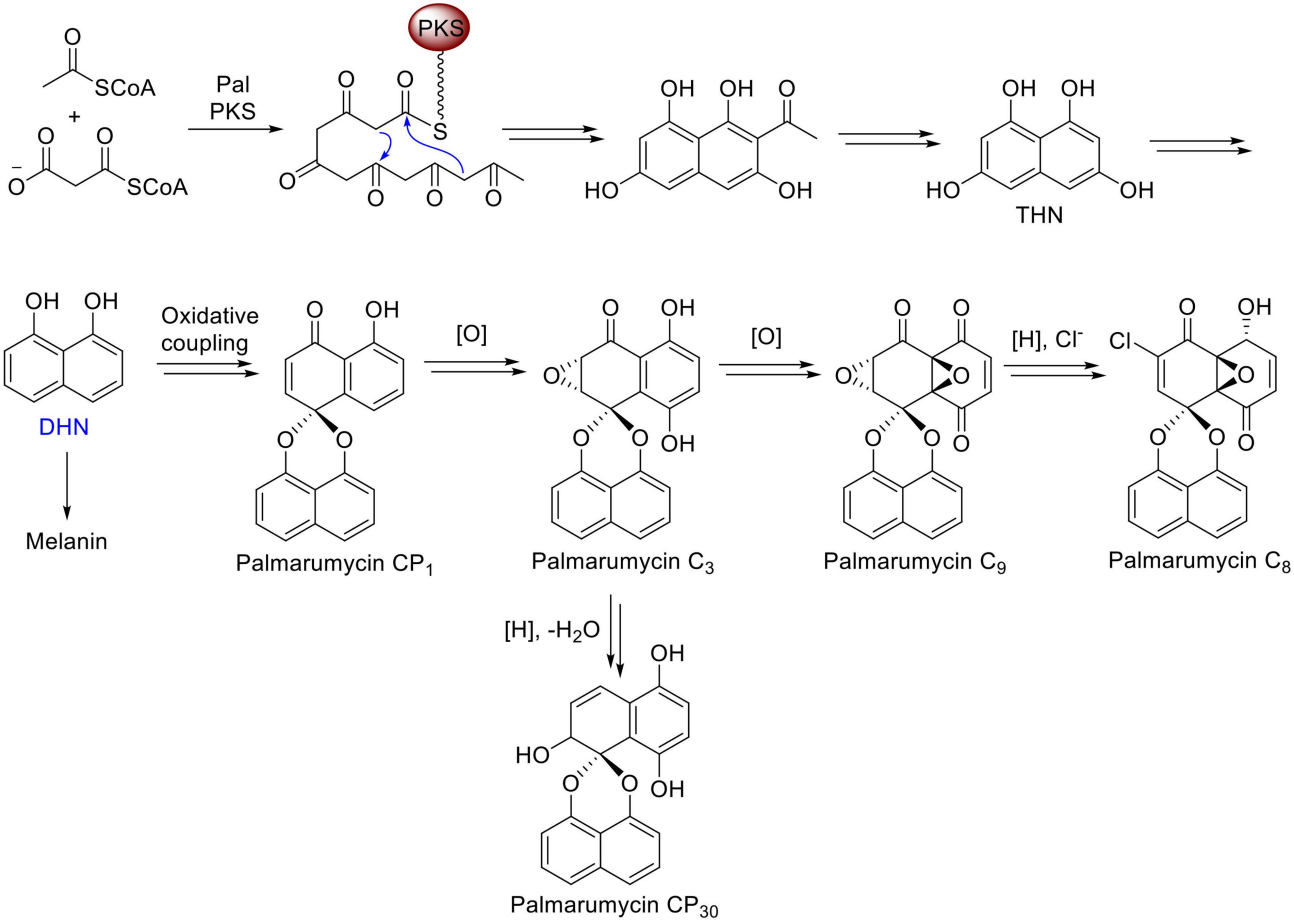
Proposed biosynthetic routes to melanin, palmarumycin C_8_, and palmarumycin CP_30_ from their shared precursor DHN in *Lophiotrema* sp. F6932.

The *PAL* gene cluster in *Lophiotrema* sp. F6932 shows high similarity to the melanin biosynthetic gene cluster from phytopathogenic fungus *Bipolaris oryzae* based on antiSMASH analysis results*. Bipolaris oryzae* produces melanin *via* the 1,8-dihydroxynaphthalene (DHN) pathway using acetate as a precursor (Moriwaki et al., 2004). Wild-type *Lophiotrema* sp. F6932 likewise is a heavily melanized fungus and its growth in different melanin synthesis inhibitors reveals that, similar to *B. oryzae,* it produces melanin *via* the DHN and not the DOPA pathway (Figure 4). *Lophiotrema* sp. F6932 produces spirobisnaphthalene palmarumycin C_8_ among other analogs such as palmarumycin CP_30_ (Gakuubi et al., 2022). Significantly, the biosynthesis of palmarumycins is proposed to involve phenolic oxidative dimerization of DHN, the same precursor that is involved in melanin biosynthesis in many filamentous fungi (Bode and Zeeck, 2000; Mafezoli et al., 2018). We, therefore, predicted the involvement of *PAL* in the biosynthesis of palmarumycins in addition to melanin in *Lophiotrema* sp. F6932. To confirm this, we employed CRISPR/Cas9 genome editing system for targeted deletion of the KS domain in *PAL* followed by homology-directed repair using hygromycin resistance gene cassette. The KS domains catalyze polyketide chain elongation *via* decarboxylative Claisen condensation of acyl and malonyl (Robbins et al., 2016; Yun et al., 2020). The resultant *Lophiotrema* sp. F6932 deletion mutants were no longer able to produce palmarumycins or other related compounds, confirming that *PAL* is responsible for the biosynthesis of this group of compounds. Moreover, the mutants were similarly unable to produce melanin when grown in PDB confirming that indeed, DHN is the immediate precursor for the biosynthesis of both palmarumycins and melanin (Mafezoli et al., 2018). This study thus shows that deletion of *PAL* KS domain rather than the entire polyketide synthases is sufficient to disrupt the genes involved in the biosynthesis of these compounds. Because the biosynthesis of dimeric pentaketide spirobisnaphthalenes such as palmarumycins that involves the phenolic oxidative coupling of DHN has been well-documented, we, therefore, propose the biosynthetic routes to melanin, palmarumycin C_8_, and palmarumycin CP_30_ from their shared precursor DHN in *Lophiotrema* sp. F6932 (Figure 7).

## Conclusion

Genetic engineering through homologous recombination (HR) has been widely used in fungal functional genomic studies. However, the efficiency of HR repair in many filamentous fungi has been found to be generally low. CRISPR-Cas9 ribonucleoprotein-mediated genome editing, a system that employs *in vitro*-assembled dual Cas9 ribonucleoprotein, has recently gained traction in different areas of fungal studies, such as in the characterization of secondary metabolite gene clusters and pathogenesis among other cellular processes. Using this approach, we have established an efficient CRISPR-Cas9 genome editing system for *Lophiotrema* sp. F6932 which we have employed to validate the polyketide synthases (*PAL*) involved in palmarumycin and melanin biosynthesis in this fungal strain. To achieve this, we applied *in vitro*-assembled Cas9 RNP to induce targeted deletion of ketosynthase (KS) domain of the *PAL* followed by homology-directed repair with hygromycin resistance cassette flanked by 50 bp of homology arms on both sides of the DSBs. The resultant deletion mutants displayed different phenotypes to that of the wild-type strain; they had different colony morphologies and were no longer able to produce palmarumycins or melanin. This confirms that *PAL* is the gene cluster responsible for palmarumycin and melanin biosynthesis in *Lophiotrema* sp. F6932. To the best of our knowledge, this is the first report of successful CRISPR-Cas9 mediated gene editing of a fungus belonging to *Lophiotrema* genus. This study therefore paves the way for implementing a similar approach in characterization of other BGCs of interest in this largely understudied fungal strain among other uncommon filamentous fungi.

## Data availability statement

The datasets presented in this study can be found in online repositories. The names of the repository/repositories and accession number(s) can be found in the article/Supplementary material.

## Author contributions

SN and MG conceptualized and planned the study. MG performed the knockout experiments. CL, G-LM, and Z-XL performed bioinformatics analyses. MM performed phylogenetic analyses. KC, MW, and YK performed the chemical analyses. All the authors contributed to the preparation of the manuscript and approved the submitted version.

## Funding

This work was funded by the Singapore Institute of Food and Biotechnology Innovation, Agency for Science, Technology, and Research (A*STAR), Singapore, through a Singapore Integrative Biosystems and Engineering Research (SIBER) Grant (#21719).

## Conflict of interest

The authors declare that the research was conducted in the absence of any commercial or financial relationships that could be construed as a potential conflict of interest.

## Publisher’s note

All claims expressed in this article are solely those of the authors and do not necessarily represent those of their affiliated organizations, or those of the publisher, the editors and the reviewers. Any product that may be evaluated in this article, or claim that may be made by its manufacturer, is not guaranteed or endorsed by the publisher.

## Footnotes

^1^ https://blast.ncbi.nlm.nih.gov/Blast.cgi
