# Supplementary material for: CRISPR/Cas9 RNP-assisted validation of palmarumycin biosynthetic gene cluster in Lophiotrema sp. F6932
Source: Front Microbiol. 2022 Sep 29;13:1012115. doi: 10.3389/fmicb.2022.1012115 (PMC9556985; doi:10.3389/fmicb.2022.1012115)
Supplement: Supplementary file 1 [file Data_Sheet_1.PDF]

Supplementary Material

CRISPR/Cas9 RNP-assisted validation of palmarumycin biosynthetic gene cluster in *Lophiotrema* sp. F6932

Martin Muthee Gakuubi, Kuan Chieh Ching, Madhaiyan Munusamy, Mario Wibowo, Lim Chun Teck, Guang-Lei Ma, Zhao-Xun Liang, Yoganathan Kanagasundaram and Siew Bee Ng*

Abstract: *Lophiotrema* is a genus of ascomycetous fungi within the family *Lophiotremataceae*. Members of this genus have been isolated as endophytes from a wide range of host plants and also from plant debris within terrestrial and marine habitats, where they are thought to function as saprobes. *Lophiotrema* sp. F6932 was isolated from white mangrove (*Avicennia officinalis*) in Pulau Ubin Island, Singapore. Crude extracts from the fungus exhibited strong antibacterial activity, and bioassay-guided isolation and structure elucidation of bioactive constituents led to the isolation of palmarumycin C_8 and its new analogue palmarumycin CP_{30}. Whole genome sequencing analysis resulted in the identification of a putative type 1 iterative PKS (PAL) predicated to be involved in the biosynthesis of palmarumycins. To verify the involvement of PAL in the biosynthesis of these compounds, we employed ribonucleoprotein (RNP)-mediated CRISPR-Cas9 to induce targeted deletion of the ketosynthase (KS) domain in PAL. Double strand breaks (DSBs) upstream and downstream of the KS domain was followed by homology directed repair (HDR) with a hygromycin resistance cassette flanked by a 50 bp of homology on both sides of the DSBs. The resultant deletion mutants displayed completely different phenotypes compared to the wild-type strain, as they had different colony morphology and were no longer able to produce palmarumycins or melanin. This study therefore, confirms the involvement of PAL in the biosynthesis of palmarumycins, and paves the way for implementing similar approach in the characterization of other gene clusters of interest in this largely understudied fungal strain.

Table S1. Summary of antiSMASH biosynthetic gene cluster predictions for *Lophiotrema* sp. F6932 genome.

Region	Type	From	To	Most similar known cluster	Similarity (%)
Region 1.1	Unknown	116,571	240,321		
Region 1.2	Unknown	308,238	349,085		
Region 1.3	Unknown	432,693	634,304		
Region 1.4	Unknown	1,065,116	1,143,482		
Region 1.5	NRPS	1,171,515	1,281,703	Aspercryptins	13
Region 1.6	T1PKS	1,381,986	1,428,072	(-)-Mellein	100
Region 1.7	T1PKS	1,870,935	1,938,884	4-epi-15-epi-brefeldin A	20
Region 1.8	NRPS	2,447,033	2,661,264	Aflatoxin G1	8
Region 1.9	Unknown	2,671,639	2,772,419		
Region 1.10	T1PKS	2,777,472	2,944,112		
Region 1.11	T3PKS	2,962,163	3,016,966		
Region 1.12	Unknown	3,515,400	3,587,309		
Region 1.13	Unknown	3,618,514	3,705,836		
Region 1.14	Unknown	4,160,513	4,246,489		
Region 1.15	Unknown	4,446,173	4,491,368		
Region 1.16	Unknown	4,577,627	4,632,326		
Region 1.17	Unknown	4,789,180	4,857,556		
Region 1.18	NRPS	5,222,761	5,281,732		
Region 2.1	T1PKS	255,128	450,350		
Region 2.2	Unknown	880,154	1,127,586	Iso-A82775C	16
Region 2.3	Unknown	1,372,944	1,409,598		
Region 2.4	Unknown	1,645,613	1,705,296		
Region 2.5	Unknown	1,714,243	1,775,793		
Region 2.6	Unknown	1,793,048	1,889,962		
Region 2.7	Unknown	1,932,646	1,995,669		
Region 2.8	T1PKS	2,061,057	2,134,556		
Region 2.9	T1PKS	2,281,707	2,394,046		
Region 2.10	Unknown	2,395,946	2,487,387		

Region	Type	From	To	Most similar known cluster	Similarity (%)
Region 2.11	Terpene	2,934,952	3,071,936		
Region 2.12	Unknown	3,347,115	3,422,145		
Region 3.1	Unknown	30,836	143,205		
Region 3.2	Terpene	418,470	515,527		
Region 3.3	Unknown	1,705,730	1,762,477		
Region 3.4	Terpene	1,822,521	1,844,145	Squalestatin S1	40
Region 3.5	Unknown	2,139,179	2,281,614	Communesin A-H	25
Region 3.6	Unknown	2,283,887	2,395,321		
Region 3.7	Unknown	2,549,618	2,613,325		
Region 3.8	Unknown	2,793,702	2,881,126		
Region 4.1	Unknown	457,215	474,184		
Region 4.2	Unknown	483,298	495,204		
Region 4.3	T1PKS	566,832	680,094		
Region 4.4	Unknown	751,992	810,512		
Region 4.5	Unknown	860,351	901,146		
Region 4.6	Unknown	1,045,540	1,176,938		
Region 4.7	Unknown	1,641,587	1,681,905		
Region 4.8	Unknown	2,248,215	2,303,379		
Region 4.9	Unknown	2,692,134	2,731,868		
Region 5.1	Unknown	840,861	866,812		
Region 5.2	NRPS-like	967,342	1,009,332		
Region 5.3	Terpene	1,360,887	1,380,249		
Region 5.4	Unknown	1,781,411	1,822,589		
Region 5.5	Unknown	1,873,117	2,011,129		
Region 5.6	NRPS-like	2,183,662	2,287,559	Chrysoxanthone A-C	13
Region 5.7	Unknown	2,305,795	2,330,488		
Region 5.8	Terpene	2,466,396	2,487,725		
Region 6.1	Unknown	6,374	86,363		
Region 6.2	Unknown	231,336	294,816		
Region 6.3	Unknown	331,211	432,282		
Region 6.4	Unknown	858,769	890,900		
Region 6.5	Unknown	1,502,836	1,549,569		
Region 6.6	Unknown	1,884,513	1,948,799		

Region	Type	From	To	Most similar known cluster	Similarity (%)
Region 7.1	NRPS	2,768	32,737		
Region 7.2	Unknown	35,688	128,694		
Region 7.3	NRPS	226,783	282,973		
Region 7.4	Unknown	714,690	881,396		
Region 7.5	Unknown	1,566,916	1,662,432		
Region 8.1	NRPS-like	14,443	100,700		
Region 8.2	Unknown	784,127	829,974		
Region 8.3	Unknown	1,077,203	1,211,982		
Region 8.4	Unknown	1,377,391	1,417,287		
Region 8.5	NRPS, terpene	1,638,249	1,748,058	Dimethylcoprogen	100
Region 9.1	Unknown	124,754	134,104		
Region 9.2	Unknown	540,894	675,828		
Region 9.3	NRPS-like	700,151	760,776		
Region 9.4	Unknown	1,303,045	1,403,301		
Region 10.1	Unknown	6,883	81,986		
Region 10.2	Unknown	653,390	683,976	Huperzine A	53
Region 10.3	Unknown	1,335,018	1,506,891	Communesin A-H	25
Region 10.4	Unknown	1,571,370	1,634,597		
Region 10.5	Unknown	1,672,504	1,727,169		
Region 11.1	NRPS-like	250,006	293,587		
Region 11.2	T1PKS	545,899	695,482	Melanin	100
Region 11.3	Unknown	868,843	882,655		
Region 11.4	Unknown	1,105,572	1,182,959		
Region 11.5	T1PKS	1,497,182	1,551,787		
Region 12.1	NRPS-like	99,563	291,320		
Region 12.2	Unknown	1,243,504	1,290,587		
Region 12.3	Unknown	1,404,586	1,418,801		
Region 12.4	Unknown	1,422,933	1,485,361		
Region 13.1	Terpene	113,501	159,214	Clavaric acid	100
Region 13.2	Unknown	235,017	289,444		
Region 13.3	Unknown	370,195	418,346		
Region 13.4	NRPS-like	420,025	467,503		
Region 13.5	Unknown	528,284	613,438		

Region	Type	From	To	Most similar known cluster	Similarity (%)
Region 13.6	NRPS	1,039,677	1,184,387	KK-1	20
Region 14.1	Unknown	10,225	82,288		
Region 14.2	Unknown	457,583	566,897		
Region 14.3	Unknown	854,261	982,390		
Region 14.4	Unknown	991,694	1,113,490		
Region 14.5	T1PKS	1,206,100	1,269,420		
Region 14.6	Unknown	1,290,264	1,329,360		
Region 15.1	T1PKS	186,603	370,274		
Region 15.2	T1PKS	824,900	958,669		
Region 15.3	Unknown	1,139,387	1,175,056		
Region 15.4	Unknown	1,280,373	1,318,193		
Region 16.1	T1PKS	238,970	393,091		
Region 16.2	Unknown	471,470	607,368		
Region 16.3	Unknown	921,512	989,845		
Region 16.4	Unknown	1,127,976	1,185,333		
Region 16.5	Unknown	1,194,854	1,263,311		
Region 17.1	Unknown	21,987	144,376		
Region 17.2	Unknown	145,953	270,216		
Region 17.3	Unknown	341,735	380,902		
Region 17.4	Unknown	471,775	596,600		
Region 17.5	NRPS-like	747,673	830,967		
Region 17.6	Unknown	1,053,176	1,115,067		
Region 17.7	T1PKS	1,117,243	1,191,476		

Table S2. *Lophiotremataceae* family members used in the phylogenetic analyses

**Species**	**Strain**	**18S**	**ITS**	**28S**	**RBP2**	**TEF**
***Lophiotrema* sp. F6932**	**F6932**	**ON723936**	**OM791904**	**ON723938**	**ON736762**	**ON736763**
Atrocalyx acutisporus	HHUF 30504	NG_061269.1	NR_153586.1	NG_057141.1	LC194423.1	LC194386.1
Atrocalyx bambusae	MFLU 11-0150	NG_063622.1	NR_153559.1	NG_057116.1	KX672161.1	KX672162.1
Atrocalyx lignicola	CBS 122364	NG_062149.1	NR_153587.1	MH874736.1	LC194424.1	LC194387.1
Crassimassarina macrospora	HHUF 29084	NG_061270.1	NR_153942.1	NG_059030.1	LC194426.1	LC194389.1
Crassiparies quadrisporus	HHUF 30409	NG_061267.1	NR_148185.1	NG_059028.1	-	-
Cryptoclypeus oxysporus	HHUF 30507	NG_062427.1	NR_153943.1	NG_059031.1	LC194427.1	LC194390.1
Cryptoclypeus ryukyuensis	HHUF 30509	NG_061271.1	NR_153944.1	NG_059032.1	LC194429.1	LC194392.1
Cryptocoryneum akitaense	HHUF 30477	NG_065115.1	NR_153935.1	NG_059033.1	LC194430.1	LC096136.1
Cryptocoryneum brevicondensatum	HHUF 30478	NG_065116.1	NR_153936.1	NG_059034.1	LC194431.1	LC096137.1
Cryptocoryneum congregatum	HHUF 30479	NG_065117.1	NR_153937.1	NG_069470.1	LC194435.1	LC096141.1
Cryptocoryneum japonicum	HHUF 30482	NG_065118.1	NR_153938.1	NG_059035.1	LC194438.1	LC096144.1
Cryptocoryneum longicondensatum	HHUF 30486	NG_065119.1	NR_153939.1	NG_069471.1	LC194442.1	LC096148.1
Cryptocoryneum paracondensatum	HHUF 30489	NG_065120.1	NR_153940.1	*NG_069472.1*	LC194445.1	LC096151.1
Cryptocoryneum pseudorilstonei	CBS 113641	NG_063082.1	NR_153941.1	NG_059036.1	LC194446.1	LC096152.1
Ernakulamia krabiensis	MFLUCC 18-0237	NG_065780.1	NR_163342.1	NG_066314.1	MK434872.1	MK360053.1
Galeaticarpa aomoriensis	HHUF 30505	NG_061272.1	NR_154104.1	NG_059809.1	LC194448.1	LC194393.1
Lophiotrema eburnoides	HHUF 30079	NG_065109.1	NR_138014.1	NG_059801.1	LC194458.1	LC194403.1
Lophiotrema fallopiae	HHUF 30506	NG_065113.1	NR_155339.1	NG_059808.1	LC194459.1	LC194404.1
Lophiotrema mucilaginosis	HMAS 255437	NG_067675.1	NR_164039.1	NG_066426.1	MH822892.1	MH822893.1
Pseudocryptoclypeus yakushimensis	HHUF 30503	NG_061273.1	NR_154379.1	NG_059810.1	LC194472.1	LC194417.1
Pseudolophiotrema elymicola	HHUF 28984	NG_061274.1	NR_154380.1	NG_059811.1	LC194473.1	LC194418.1

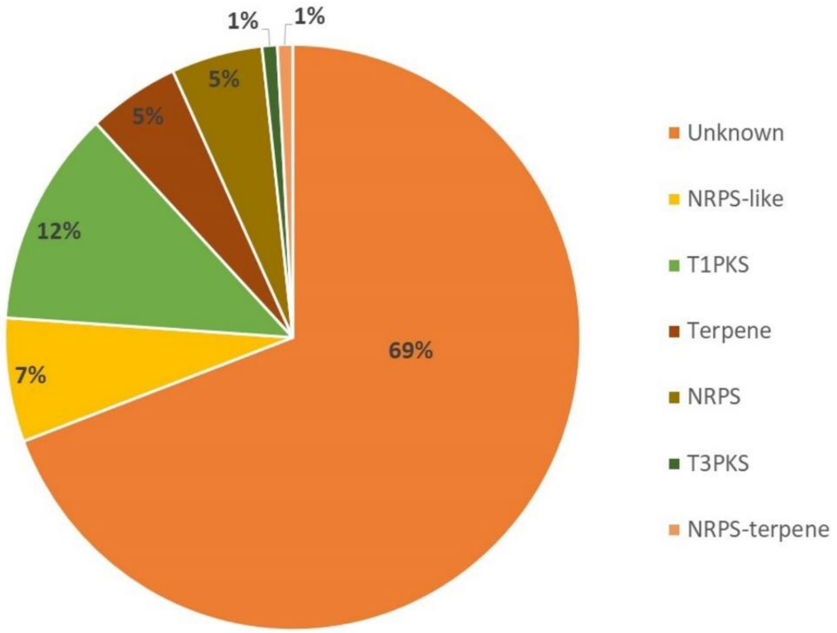

Figure S1. A summary of the classes of secondary metabolite gene clusters in *Lophiotrema* sp. F6932 genome as predicted by antiSMASH.

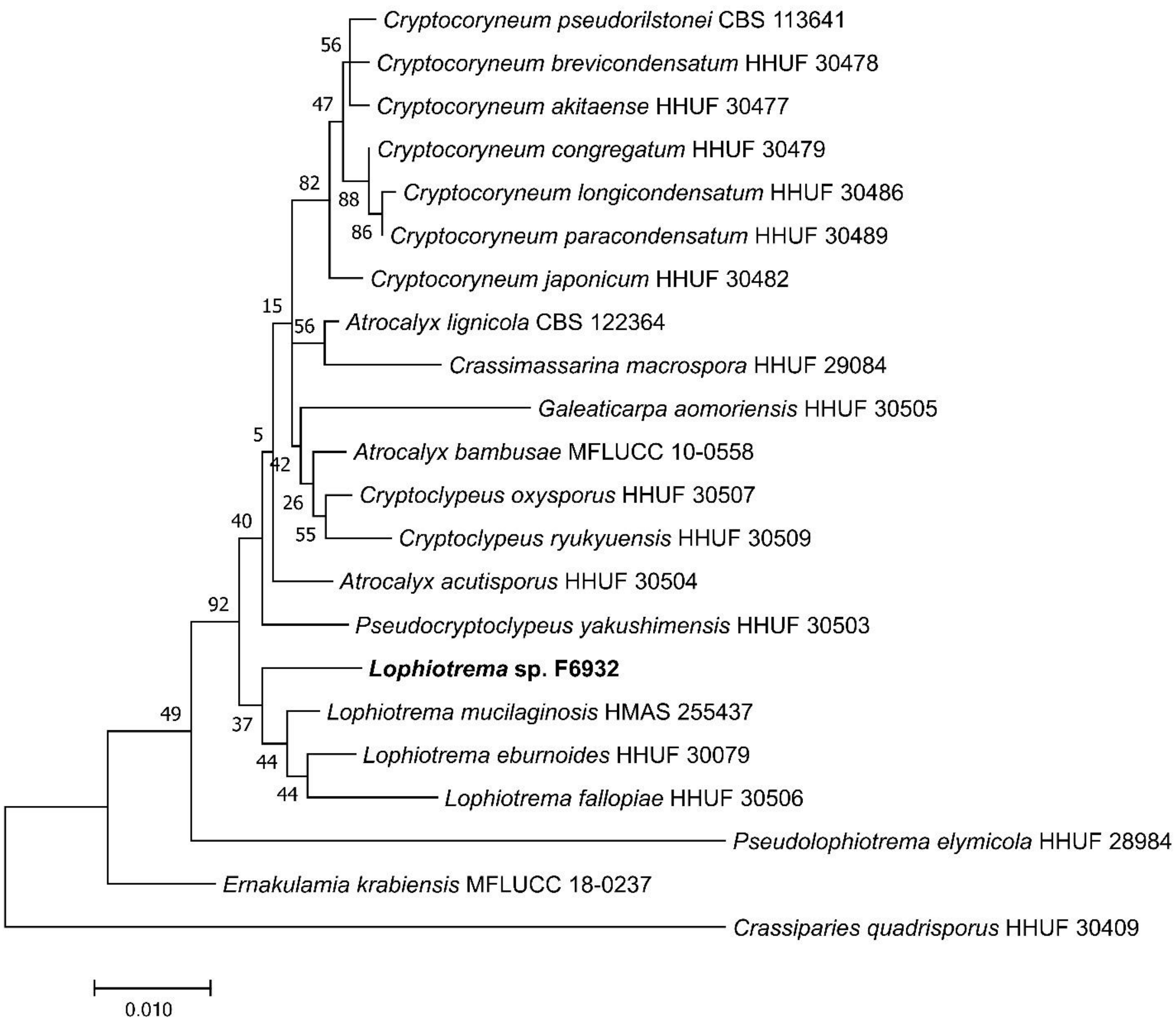

Figure S2. Molecular phylogenetic analysis by maximum likelihood method showing members of the family *Lophiotremataceae* with *Crassiparies quadrisporus* HHUF 30409 as the outgroup (1000 bootstrap replications). The evolutionary history was inferred using maximum likelihood method based on the Tamura-Nei model (Tamura and Nei, 1993). The tree with the highest log likelihood (-5033.39) is shown. The percentage of trees in which the associated taxa clustered together is shown next to the branches. Initial tree(s) for the heuristic search were obtained automatically by applying Neighbor-Join and BioNJ algorithms to a matrix of pairwise distances estimated using the Maximum Composite Likelihood (MCL) approach, and then selecting the topology with superior log likelihood value. The tree is drawn to scale, with branch lengths measured in the number of substitutions per site. The analysis involved 22 nucleotide sequences. Codon positions included were 1st+2nd+3rd+Noncoding. All positions containing gaps and missing data were eliminated. There were a total of 1788 positions in the final dataset. Evolutionary analyses were conducted in MEGA7 (Kumar et al., 2016). All the sequences used in this analysis are listed in **Table S2.**

frontiers

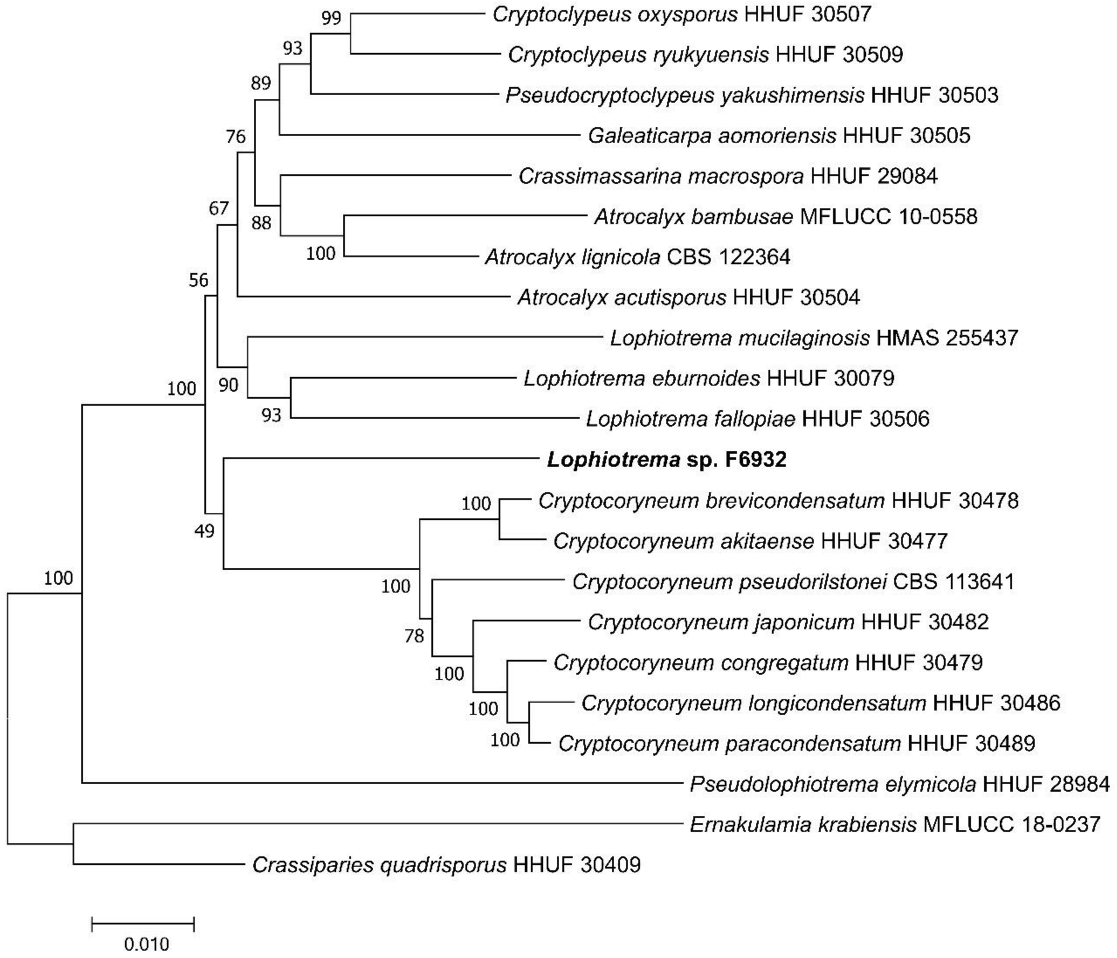

Figure S3. Molecular phylogenetic analysis by neighbor-joining method showing members of the family *Lophiotremataceae* with *Crassiparies quadrisporus* HHUF 30409 as the outgroup. The evolutionary history was inferred using the neighbor-joining method (Saitou and Nei, 1987). The optimal tree with the sum of branch length = 0.54925145 is shown. The percentage of replicate trees in which the associated taxa clustered together in the bootstrap test (1000 replicates) are shown next to the branches (Felsenstein, 1985). The tree is drawn to scale, with branch lengths in the same units as those of the evolutionary distances used to infer the phylogenetic tree. The evolutionary distances were computed using the maximum composite likelihood method (Tamura et al., 2004) and are in the units of the number of base substitutions per site. The analysis involved 22 nucleotide sequences. Codon positions included were 1st+2nd+3rd+Noncoding. All ambiguous positions were removed for each sequence pair. There were a total of 4465 positions in the final dataset. Evolutionary analyses were conducted in MEGA7 (Kumar et al., 2016). All the sequences used in this analysis are listed in **Table S2.**

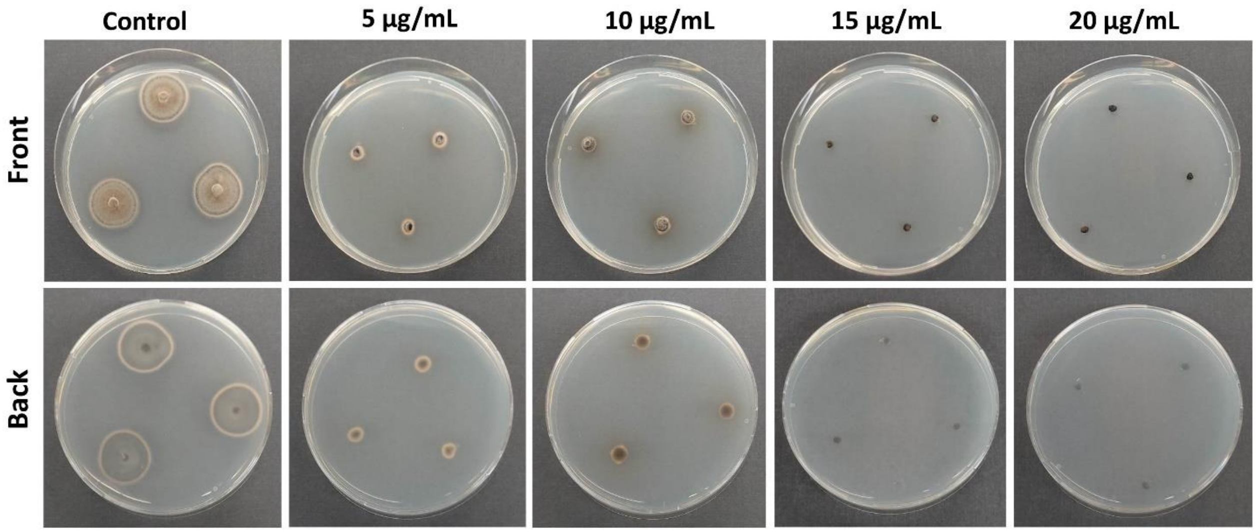

Figure S4. Sensitivity of wild type *Lophiotrema* sp. F6932 grown in PDA with different concentrations of hygromycin B after 14 days.

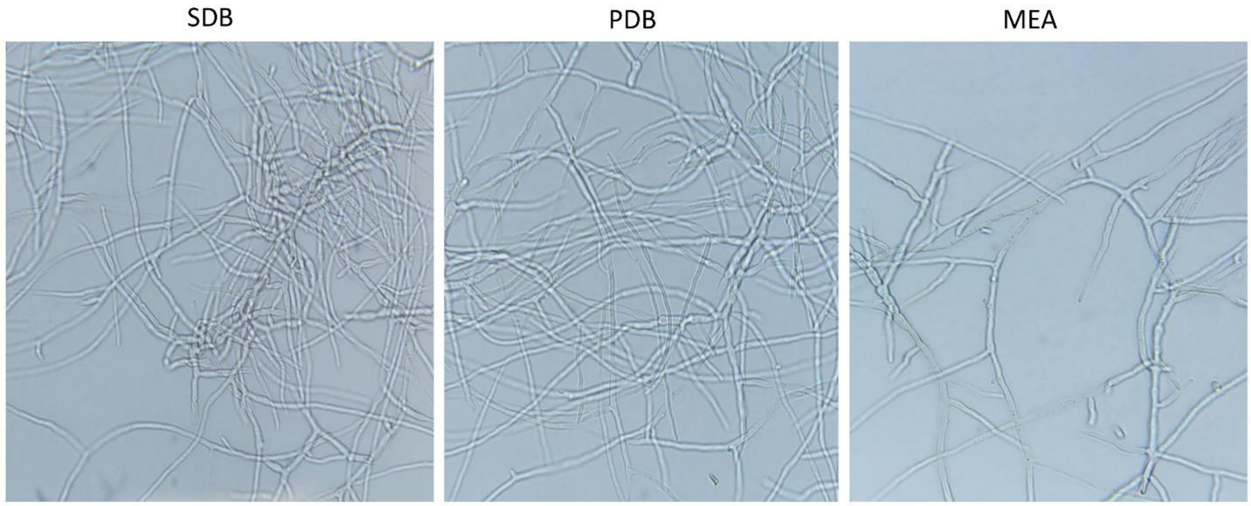

Figure S5. *Lophiotrema* sp. F6932 mycelial growth on Sabouraud dextrose broth (SDB), potato dextrose broth (PDB) and malt extract broth (MEB) after 4 days.

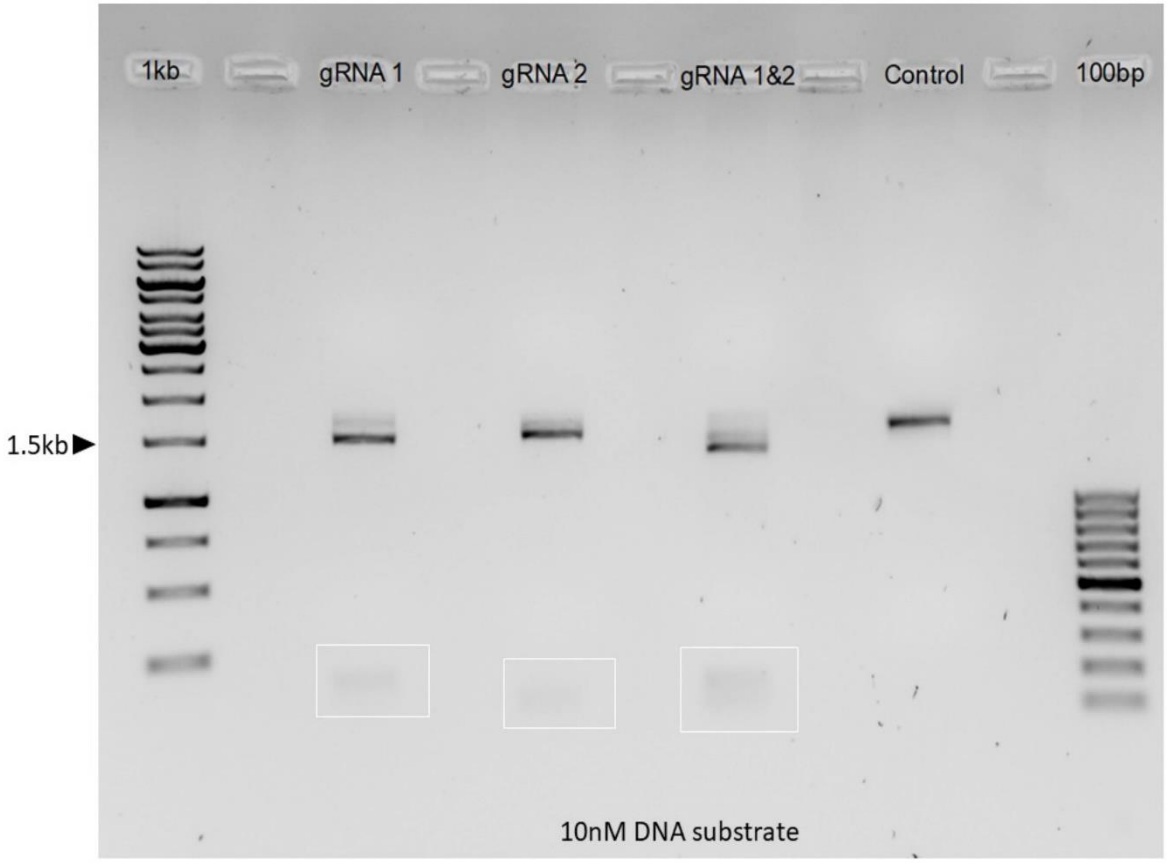

Figure S6. sgRNA-mediated *in vitro* cleavage of the target region. 10 nM of 1664 bp PCR fragment encompassing ketosynthase (KS) domain of *PAL* was mixed with Cas9 enzyme and each of the two sgRNAs first individually and then together. For the negative control, a reaction with all the reagents minus the Cas9 RNP was included for comparison. Bands of lower size (<200 bp) appears as smears at the bottom of the gel photographs and have been highlighted using white rectangles and their sizes can be estimated using the 100 bp ladder.

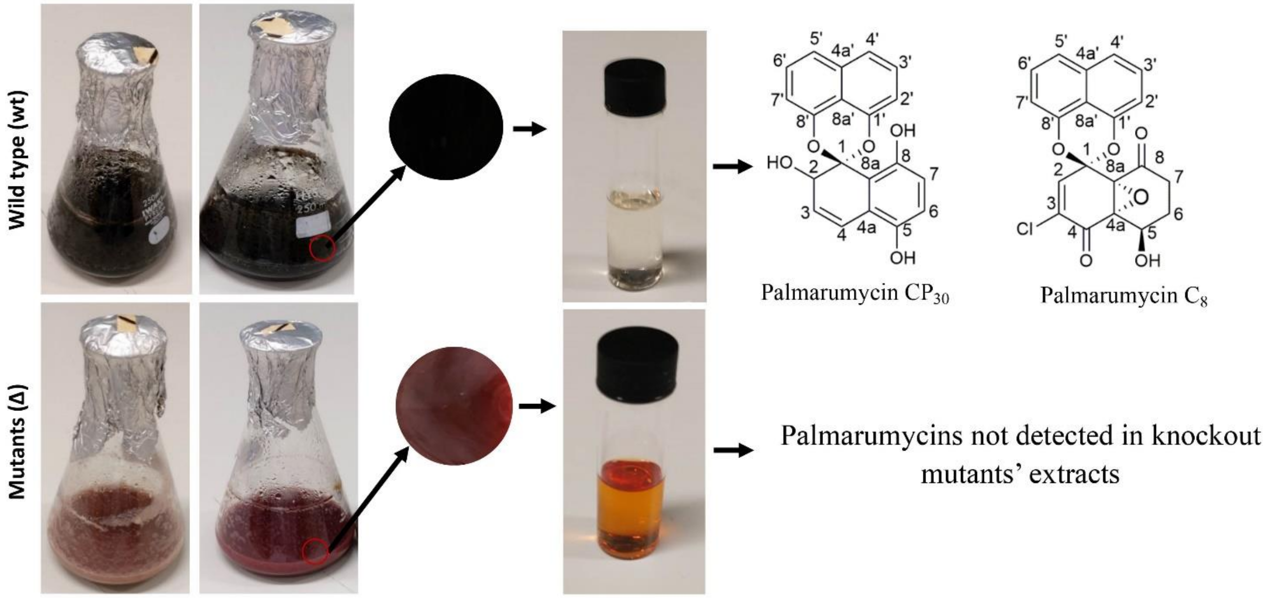

Figure S7. Differences in the coloration of crude extracts derived from wild-type and CRISPR/Cas9-induced *Lophiotrema* sp. F6932 mutants grown in CF02LB media.

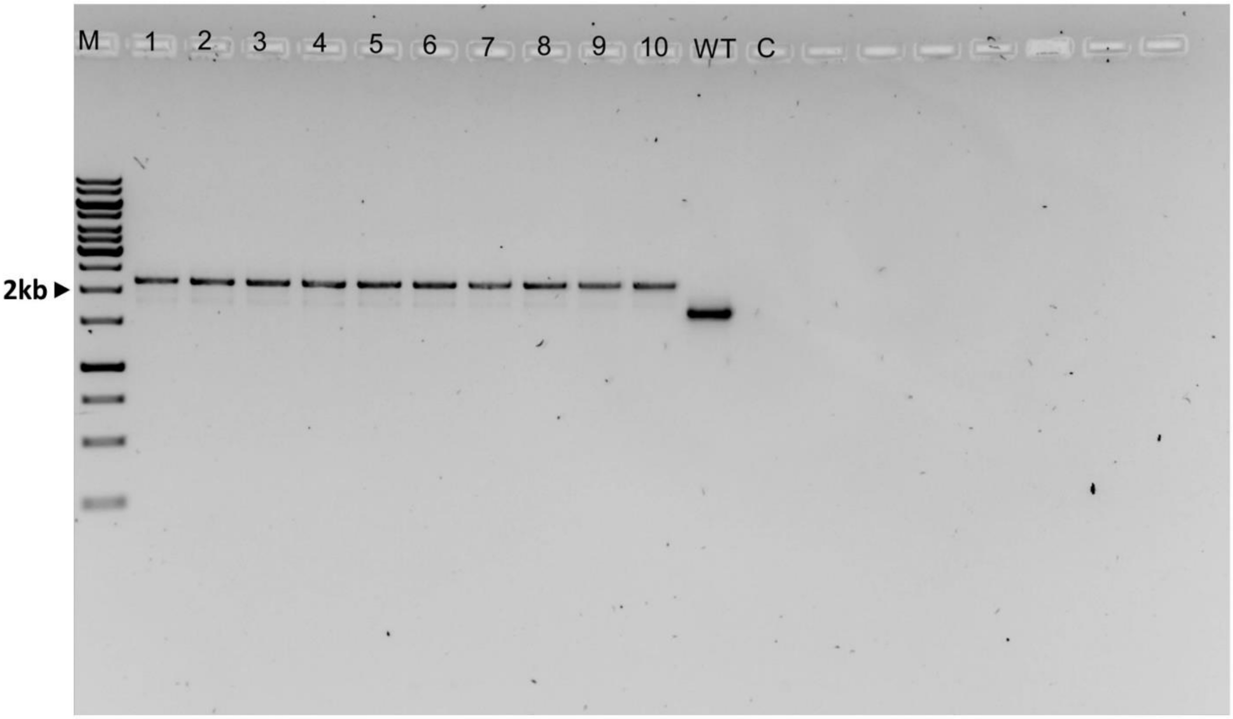

Figure S8 A. ChkF/ChkR

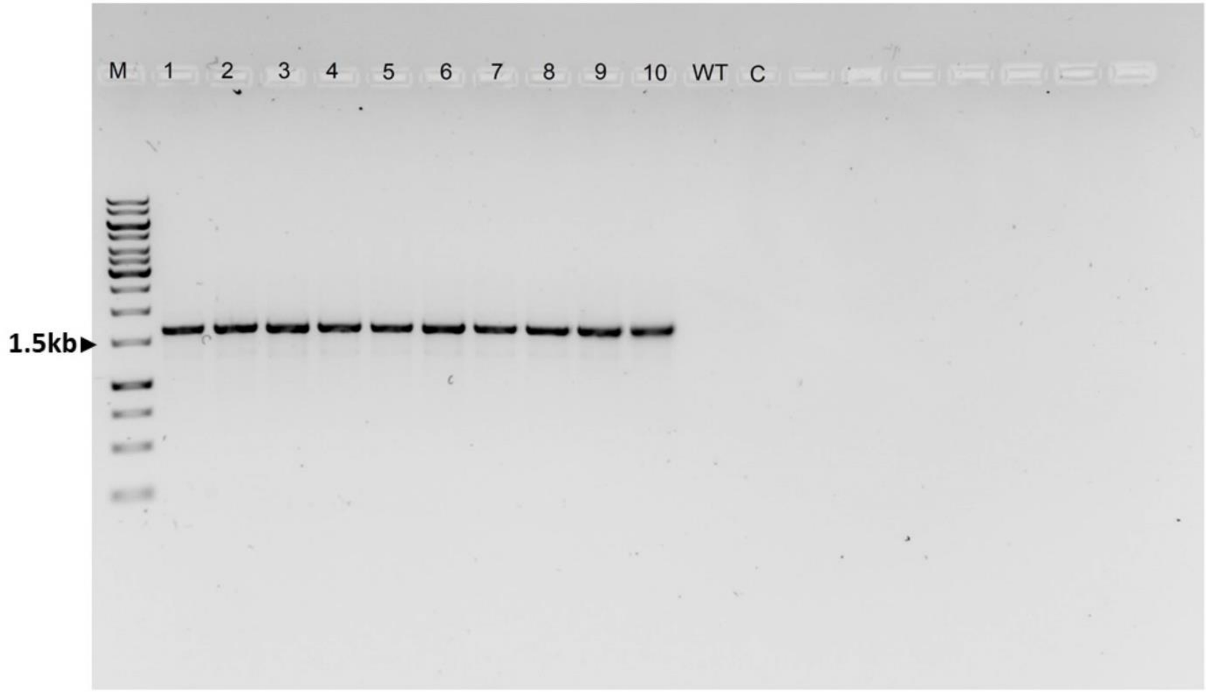

Figure S8 B. KSF1/HyR1

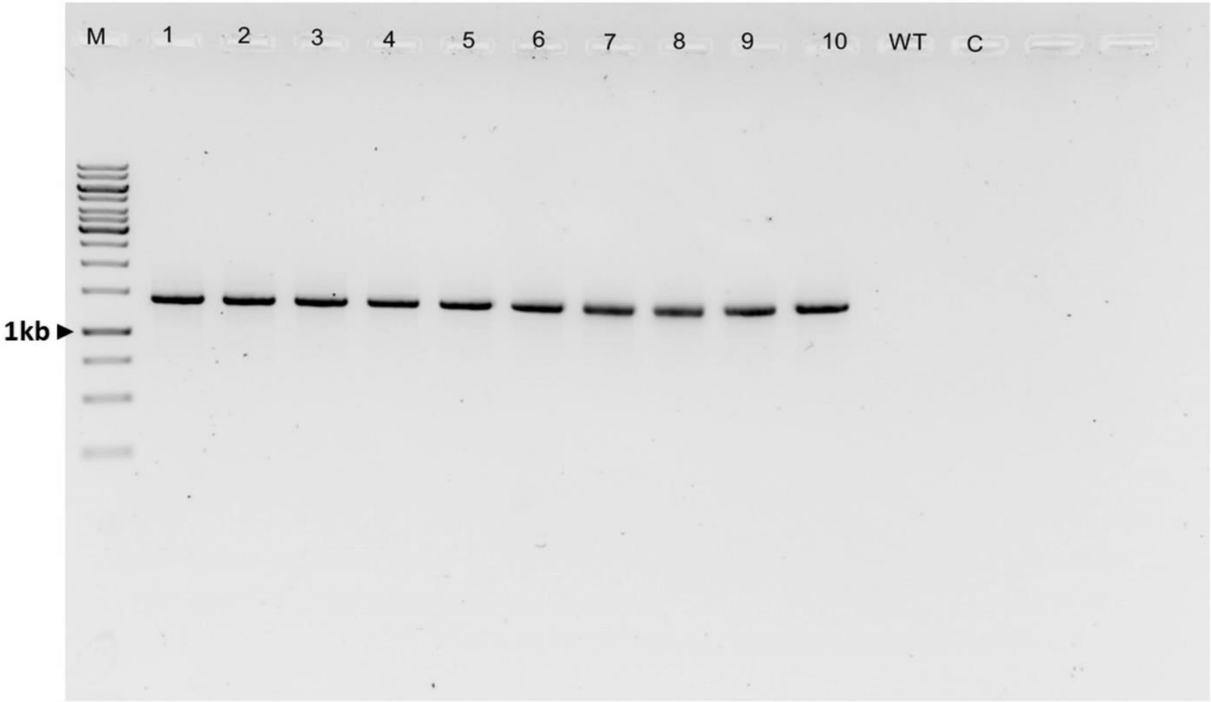

Figure S8 C. HyF1/KSR1

Figure S8. Full-length gel pictures from PCR analysis of *Lophiotrema* sp. F6932 CRISPR/Cas9-induced mutants.

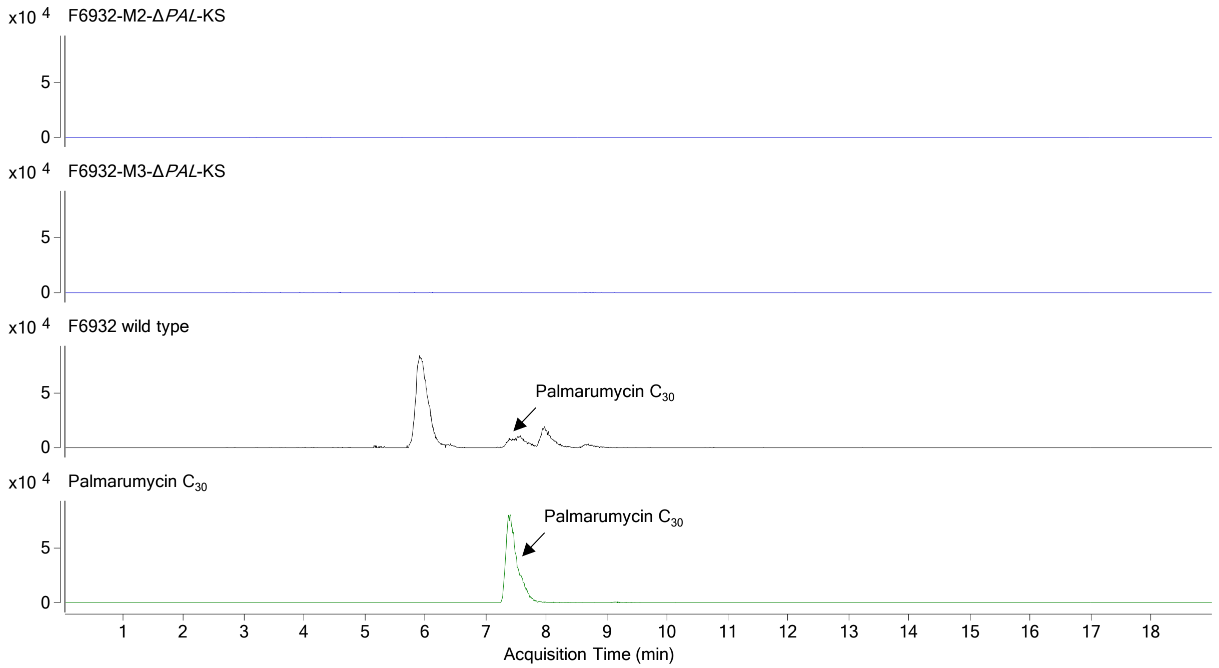

Figure S9. Extracted ion chromatograms (EIC) for palmarumycin C_{30} of cultures extracts from wild type *Lophiotrema* sp. F6932 (black line) and two arbitrarily selected Δ*PAL*-KS mutants (blue lines). Palmarumycin C_{30} (*m/z* 317.0816 ± 10 ppm, RT 7.4 min).

References

Andreasen, M., Skrede, I., Jaklitsch, W.M., Voglmayr, H., Nordén, B. (2021). Multi-locus phylogenetic analysis of lophiostomatoid fungi motivates a broad concept of *Lophiostoma* and reveals nine new species. *Persoonia* 46: 240–271. https://doi.org/10.3767/persoonia.2021.46.09.

Felsenstein, J. (1985). Confidence limits on phylogenies: an approach using the bootstrap. *Evolution*, 39:783-791.

Hashimoto, A., Matsumura, M., Hirayama, K., Tanaka, K. (2017). Revision of *Lophiotremataceae* (*Pleosporales*, *Dothideomycetes*): *Aquasubmersaceae*, *Cryptocoryneaceae*, and *Hermatomycetaceae* fam. nov. *Persoonia-Molecular Phylogeny and Evolution of Fungi*. 39:51-73. https://doi.org/10.3767/persoonia.2017.39.03

Kumar, S., Stecher, G., and Tamura, K. (2016). MEGA7: Molecular Evolutionary Genetics Analysis version 7.0 for bigger datasets. *Mol. Biol. Evol.* 33: 1870-1874. doi:10.1093/molbev/msw054.

Saitou, N. and Nei, M., 1987. The neighbor-joining method: a new method for reconstructing phylogenetic trees. *Molecular Biology and Evolution*, 4: 406-425.

Tamura K, Nei M. (1993). Estimation of the number of nucleotide substitutions in the control region of mitochondrial DNA in humans and chimpanzees. Molecular Biology and Evolution. 10:512-26.

Tamura, K., Nei, M. and Kumar, S., 2004. Prospects for inferring very large phylogenies by using the neighbor-joining method. *Proceedings of the National Academy of Sciences*, *101*:11030-11035.
